# USP8 prevents aberrant NF-κB and Nrf2 activation by counteracting ubiquitin signals from endosomes

**DOI:** 10.1083/jcb.202306013

**Published:** 2024-01-05

**Authors:** Akinori Endo, Toshiaki Fukushima, Chikage Takahashi, Hikaru Tsuchiya, Fumiaki Ohtake, Sayaka Ono, Tony Ly, Yukiko Yoshida, Keiji Tanaka, Yasushi Saeki, Masayuki Komada

**Affiliations:** 1 Laboratory of Protein Metabolism, Tokyo Metropolitan Institute of Medical Science, Tokyo, Japan; 2 Cell Biology Center, Institute of Innovative Research, Tokyo Institute of Technology, Yokohama, Japan; 3 School of Life Science and Technology, Tokyo Institute of Technology, Yokohama, Japan; 4 Institute for Advanced Life Sciences, Hoshi University, Tokyo, Japan; 5 Molecular Cell and Developmental Biology, School of Life Sciences, University of Dundee, Dundee, UK; 6 Division of Protein Metabolism, Institute of Medical Science, The University of Tokyo, Tokyo, Japan

## Abstract

K63-linked ubiquitin chains attached to plasma membrane proteins serve as tags for endocytosis and endosome-to-lysosome sorting. USP8 is an essential deubiquitinase for the maintenance of endosomal functions. Prolonged depletion of USP8 leads to cell death, but the major effects on cellular signaling pathways are poorly understood. Here, we show that USP8 depletion causes aberrant accumulation of K63-linked ubiquitin chains on endosomes and induces immune and stress responses. Upon USP8 depletion, two different decoders for K63-linked ubiquitin chains, TAB2/3 and p62, were recruited to endosomes and activated the TAK1–NF-κB and Keap1–Nrf2 pathways, respectively. Oxidative stress, an environmental stimulus that potentially suppresses USP8 activity, induced accumulation of K63-linked ubiquitin chains on endosomes, recruitment of TAB2, and expression of the inflammatory cytokine. The results demonstrate that USP8 is a gatekeeper of misdirected ubiquitin signals and inhibits immune and stress response pathways by removing K63-linked ubiquitin chains from endosomes.

## Teaser

USP8 depletion causes aberrant accumulation of K63-linked ubiquitin chains on endosomes, where TAB2/3 and p62 are recruited, and activates NF-κB and Nrf2. The results demonstrate that USP8 is a gatekeeper of misdirected ubiquitin signals to prevent immune and stress responses.

## Introduction

Ubiquitination is a versatile and reversible posttranslational modification that triggers diverse cellular functions. Ubiquitin is conjugated to thousands of proteins in an ATP-dependent manner via a cascade of E1, E2, and E3 enzymes and can be removed by deubiquitinases (DUBs; Akimov et al., 2018; Clague et al., 2019; Hershko and Ciechanover, 1998). The functional diversity of ubiquitin modification is believed to be based on the distinct topologies of different types of ubiquitination. Substrates are modified by either monoubiquitin or polyubiquitin. In polyubiquitin chains, ubiquitin is linked through any of seven lysine residues or through the N-terminal methionine, resulting in eight possible homogeneous polyubiquitin chains (Komander and Rape, 2012). Among these, lysine-48–linked and lysine-63–linked polyubiquitin chains (K48 and K63 ubiquitin chains, respectively) are the two most abundant chain types in cells under basal conditions. K48 ubiquitin chains target substrates for proteasomal degradation, whereas K63 ubiquitin chains act as signals for endocytosis, DNA damage responses, and immune responses (Husnjak and Dikic, 2012). Although the biological significance of other homogeneous polyubiquitin chains has been investigated, they are less well characterized. The properties of different ubiquitin modifications, including linkage type and length, are recognized by distinct “ubiquitin decoders” that determine downstream ubiquitin signals, leading to specific cellular functions (Komander and Rape, 2012).

K63 ubiquitin chains play critical roles in intracellular membrane trafficking, including endocytosis and endosome–lysosome sorting (Haglund and Dikic, 2012). Steady-state or ligand-dependent ubiquitination of plasma membrane proteins is thought to rapidly induce endocytosis, and internalized plasma membrane proteins are transported to early endosomes. Ubiquitinated proteins on early endosomes are sequentially recognized by ESCRT complexes (endosomal sorting complexes required for transport-0, -Ⅰ, -Ⅱ, and -Ⅲ) and delivered to late endosomes/lysosomes where they are degraded by acid hydrolases (Williams and Urbé, 2007). With the exception of ESCRT-Ⅲ, components of each ESCRT complex have been shown to directly recognize mono-ubiquitinated and K63-linked ubiquitinated proteins via their ubiquitin-binding domains. ESCRT-mediated endosomal functions are essential for cell survival and the resulting prolonged defect in the endosomal machinery leads to cell death (Ruland et al., 2001).

In addition to being recognized by ESCRT complexes, ubiquitinated endosomal proteins undergo deubiquitination by DUBs such as USP8 under certain cellular conditions. This process results in deubiquitinated proteins escaping lysosomal degradation and being translocated to the cell surface (Clague et al., 2012). USP8 is an endosomal deubiquitinase that belongs to the ubiquitin-specific protease (USP) family. Over the past decades, two fundamental roles of USP8 in endosomal regulation have been reported. USP8 deubiquitinates internalized plasma membrane proteins such as epidermal growth factor receptor (EGFR) on endosomes and recycles them to the plasma membrane, thereby increasing their abundance (Mizuno et al., 2005). USP8 also deubiquitinates and stabilizes HRS and STAM1/2, components of the ESCRT-0 complex, which initially recognizes ubiquitinated plasma membrane proteins and primes their lysosomal sorting on endosomes (Row et al., 2006). Thus, USP8 plays critical roles in removing ubiquitinated plasma membrane proteins from endosomes by increasing recycling to the cell membrane and transport to lysosomes. Consistently, not only TSG101 knockout but also USP8 knockout in mice results in embryonic lethality (Niendorf et al., 2007; Ruland et al., 2001). The clinical relevance that hotspot mutations in the USP8 gene cause Cushing’s disease derived from a pituitary tumor supports the critical roles of USP8 in cellular functions (Reincke et al., 2015). However, few studies have investigated the major effects of USP8 depletion on cellular signaling pathways. To address this knowledge gap, we investigated the cellular responses to the transient loss of endosomal functions caused by USP8 depletion.

In this study, our proteomic screening revealed the accumulation of K63 ubiquitin chains on endosomes and the consequent induction of immune response in USP8-depleted cells. Further analysis showed that decoders for K63 ubiquitin chains, such as TAB2/3 and p62, were recruited to endosomes and consequently activated NF-κB and Nrf2 signaling pathways. These results suggest that USP8 is a gatekeeper of misdirected ubiquitin signals from endosomes.

## Results

### USP8 depletion induces abnormal accumulation of K63-linked ubiquitinated plasma membrane proteins on endosomes

**Figure 1.**
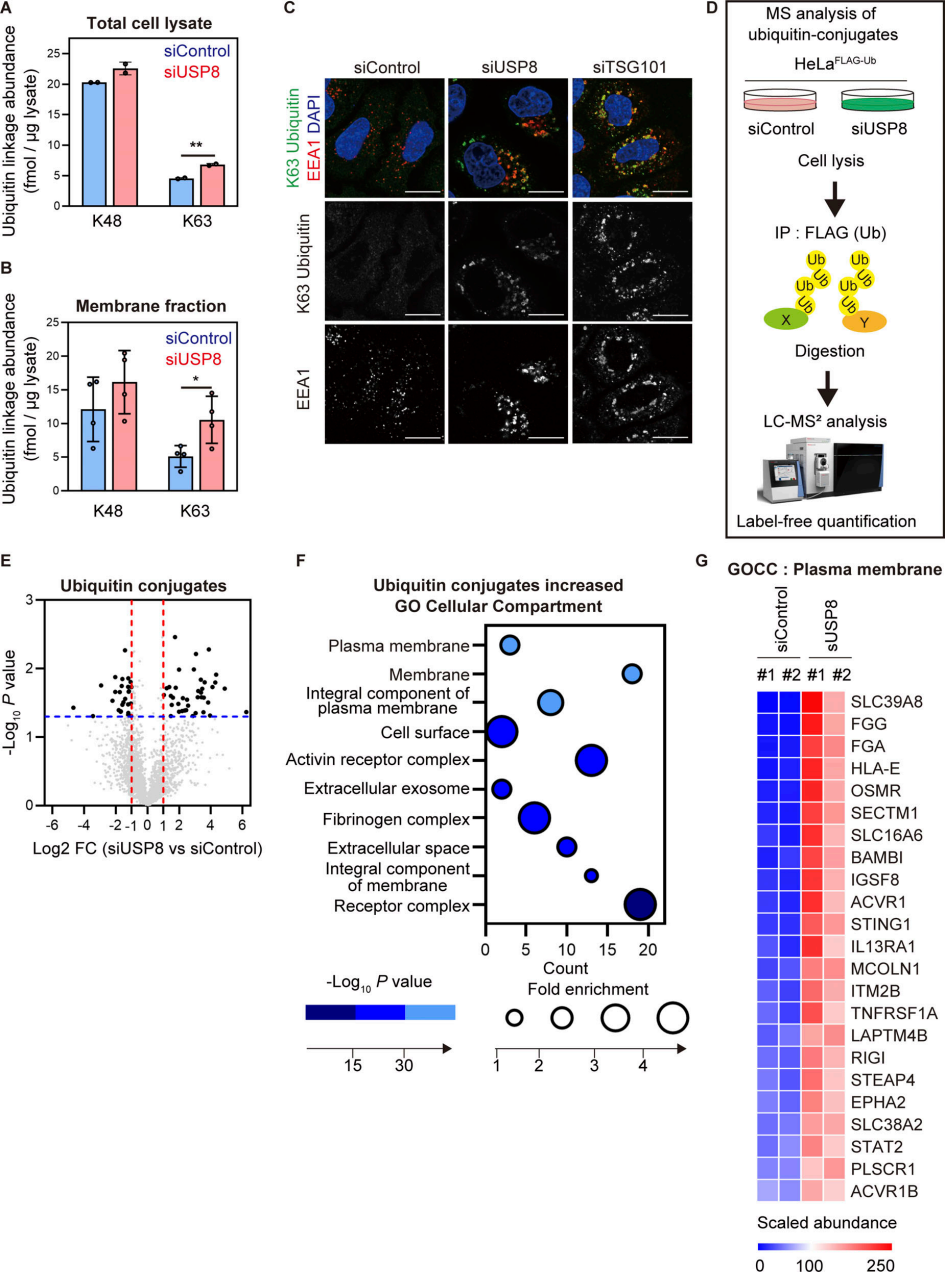
**USP8 depletion induces abnormal accumulation of K63-linked ubiquitinated plasma membrane proteins on endosomes. (A and B)** HeLa cells were transfected with the indicated siRNAs. The absolute abundance of K48 and K63 ubiquitin chains in total cell lysates (A) or membrane fractions (B) was analyzed by Ub-PRM. Error bars indicate SD (total cell lysate *n* = 2 in A, membrane fraction *n* = 4 in B). *P < 0.05 and **P < 0.01 (two-tailed Student’s *t* test). **(C)** HeLa cells transfected with the indicated siRNAs were immunostained with the indicated antibodies and DAPI. Scale bar, 20 μm. **(D)** Experimental procedures are shown (see also Materials and methods section). HeLa cells transfected with the indicated siRNAs were subjected to LC-MS/MS analysis. **(E)** The mean log_2_ FC (siUSP8/siControl) and −log_10_ P value of the ubiquitin conjugates analysis are shown on the x- and y-axis, respectively. **(F)** The bubble plot illustrating the GOCC of ubiquitinated proteins increased in USP8-depleted cells is shown (top 10 categories). **(G)** The heatmap illustrates the changes of proteins hit in the plasma membrane in F. Colors indicate the scaled intensity.

**Figure S1.**
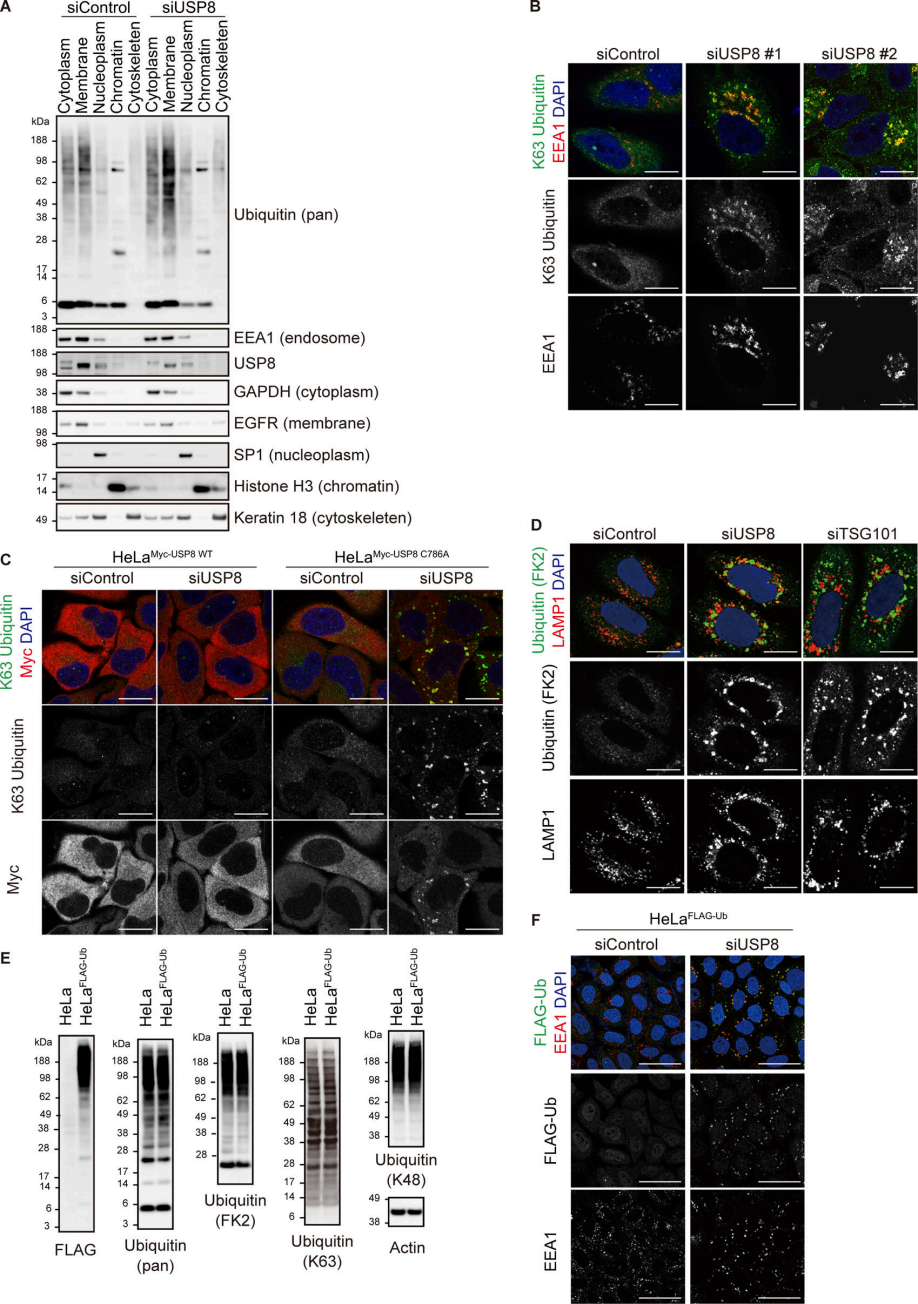
**Ubiquitin accumulation in USP8-depleted cells. (A)** HeLa cells transfected with the indicated siRNAs were subjected to subcellular fractionation. Each fraction (cytoplasm, membrane, nucleoplasm, chromatin, and cytoskeleton) was immunoblotted with the indicated antibodies. **(B)** HeLa cells transfected with the indicated siRNAs were immunostained with the indicated antibodies and DAPI. Scale bar, 20 μm. **(C)** HeLa cells stably expressing siRNA-resistant Myc-USP8 WT and C786A were transfected with the indicated siRNAs. Cells were immunostained with the indicated antibodies and DAPI. Scale bar, 20 μm. **(D)** HeLa cells transfected with the indicated siRNAs were immunostained with the indicated antibodies and DAPI. Scale bar, 20 μm. **(E)** Total cell lysates from parental HeLa cells and HeLa cells stably expressing FLAG-Ub were immunoblotted with the indicated antibodies. **(F)** HeLa cells stably expressing FLAG-Ub were transfected with the indicated siRNAs. Cells were immunostained with the indicated antibodies and DAPI. Scale bar, 50 μm. Source data are available for this figure: SourceData FS1.

First, to investigate how defects in endosomal function caused by USP8 depletion affect cellular ubiquitin flux, we quantified the absolute amount of cellular ubiquitin linkages in USP8-depleted cells using absolute quantification (AQUA) of ubiquitin (Ub) peptides by parallel reaction monitoring (PRM) (Ub-AQUA/PRM) (Tsuchiya et al., 2013). siRNA-mediated depletion of USP8 increased the cellular abundance of K63 ubiquitin chains by ∼50% (from ∼5 to ∼7.5 fmol per 1 µg of total cell lysate) compared with control cells, whereas little change was observed in the level of K48 ubiquitin chains (Fig. 1 A). We then performed crude subcellular fractionation and found that ubiquitinated proteins were preferentially increased in the membrane fractions enriched for EEA1, an early endosome marker protein, of USP8-depleted cells (Fig. S1 A). K63 ubiquitin chains were increased twofold (from ∼5 to ∼10 fmol per 1 µg of membrane proteins) in the membrane fraction of USP8-depleted cells compared with control cells (Fig. 1 B and Fig. S1 A). Since USP8 presents no linkage selectivity for ubiquitin chain cleavage (Mizuno et al., 2005; Takahashi et al., 2020), it is possible that the increase in K63 ubiquitin chains was due not only to direct substrates but also to a variety of plasma membrane proteins, including non-substrates of USP8, that underwent K63-linked ubiquitination during endocytosis and accumulated on defective endosomes. To examine whether K63 ubiquitin chains accumulated in early endosomes of the USP8-depleted cells, cells were costained with anti-K63 ubiquitin chains and anti-EEA1 antibodies. K63 ubiquitin chains were dramatically accumulated on enlarged early endosomes in both USP8- and TSG101- (ESCRT-Ⅰ) depleted cells (Fig. 1 C and Fig. S1 B). K63 ubiquitin accumulation on early endosomes in USP8-depleted cells was rescued by the add-back of wild-type (WT) USP8, but not the catalytically inactive C786A mutant, in a deubiquitinating activity-dependent manner (Fig. S1 C). In contrast, little or no ubiquitin accumulated on late endosomes or lysosomes in USP8-depleted cells (Fig. S1 D). These data clearly demonstrate that the decrease in USP8 activity causes K63 ubiquitin accumulation on early endosomes via defects in the endosomal sorting machinery, consistent with previous reports (Lobert et al., 2010; Mizuno et al., 2006; Row et al., 2006). Assuming that most of the increased K63 ubiquitin chains accumulate on endosomes, we speculate that its local concentration around endosomes can be estimated to be extremely high. The abnormal accumulation of K63 ubiquitin chains on endosomes was termed “endosomal stress” in this study.

Next, we generated HeLa cells stably expressing FLAG-tagged ubiquitin (HeLa^FLAG-Ub^). We confirmed that stably expressed FLAG-Ub diffused throughout the cells and did not affect the total amount of cellular ubiquitinated proteins and free ubiquitin (Fig. S1 E) and that FLAG-Ub conjugated proteins accumulated on endosomes in USP8-depleted HeLa^FLAG-Ub^ cells (Fig. S1 F). To characterize the ubiquitinated proteins accumulated on endosomes by endosomal stress, we performed liquid chromatography–tandem mass spectrometry (LC-MS/MS) analysis using HeLa^FLAG-Ub^ cells. FLAG-Ub conjugated proteins were purified from control and USP8-depleted HeLa^FLAG-Ub^ cells using anti-FLAG antibody and were processed for LC-MS/MS analysis with the biological duplicate (Fig. 1 D). This analysis identified 39 proteins whose ubiquitination levels were increased more than twofold (log_2_ fold change [FC] [siUSP8/siControl] > 1) with P value <0.05 (Fig. 1 E and Tables S1 and S2). We also performed a gene ontology cellular compartment (GOCC) enrichment test using the DAVID platform (https://david.ncifcrf.gov; Huang et al., 2009; Sherman et al., 2022), which showed that the ubiquitination levels of proteins categorized as plasma membrane proteins were increased in USP8-depleted cells (Fig. 1, F and G).

Collectively, these data suggest that K63-linked ubiquitinated plasma membrane proteins accumulate on defective endosomes.

### Endosomal stress induces the expression of immune-related genes

**Figure 2.**
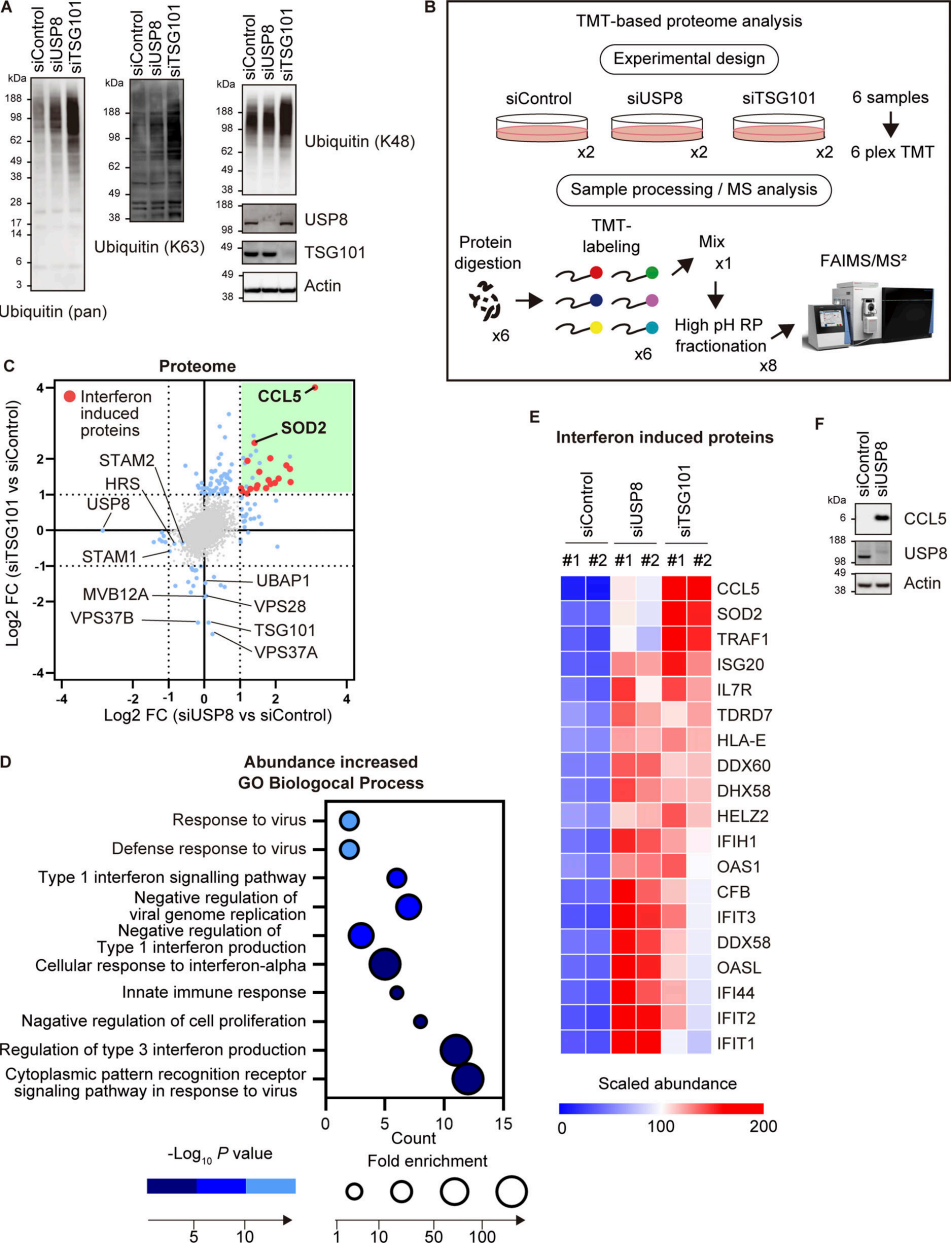
**Endosomal stress induces the expression of immune-related genes. (A)** Total cell lysates from HeLa cells transfected with the indicated siRNAs were immunoblotted with the indicated antibodies. **(B)** Experimental procedures are shown (see also Materials and methods section). HeLa cells transfected with the indicated siRNAs were subjected to LC-MS/MS analysis. **(C)** The mean Log_2_ FC (siUSP8/siControl, and siTSG101/siControl) are shown on the x- and y-axis, respectively. **(D)** The bubble plot illustrating the GO enrichment test for biological process of proteins increased in USP8-depleted cells is shown (top 10 categories). **(E)** The heatmap illustrates the changes of interferon-induced proteins increased in USP8-depleted cells. Colors indicate the scaled intensity. **(F)** Total cell lysates from HeLa cells transfected with the indicated siRNAs were immunoblotted with the indicated antibodies. Source data are available for this figure: SourceData F2.

**Figure S2.**
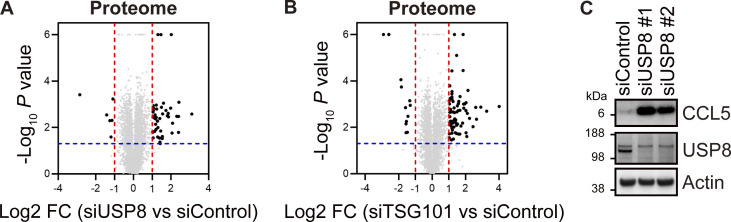
**Proteomic analysis of USP8- and TSG101-depleted cells. (A and B)** The mean Log_2_ FC (siUSP8/siControl in A and siTSG101/siControl in B) and -Log_10_ P value of the ubiquitin conjugates analysis are shown on the x- and y-axis, respectively. **(C)** Total cell lysates from HeLa cells transfected with the indicated siRNAs were immunoblotted with the indicated antibodies. Source data are available for this figure: SourceData FS2.

To characterize the dynamic changes in the proteome caused by endosomal stress, we performed Tandem Mass Tag (TMT)–based quantitative proteomics analysis of control, USP8-depleted, and TSG101-depleted cells (Fig. 2, A and B). In total, we identified 7,917 proteins and quantified 7,493 proteins among them (Fig. 2 C; Fig. S2, A and B; and Table S3). Proteins significantly upregulated in USP8- and TSG101-depleted cells showed a mild correlation and half of the proteins overlapped (Fig. 2 C). Most of these were putative interferon-induced proteins that have been reported to increase at the mRNA level (10-fold in any cells treated with any interferons, http://www.interferome.org/interferome; Rusinova et al., 2013). Note that we observed specific downregulation of the components of ESCRT-Ⅰ (i.e., VPS28, VPS37A/B, MVB12A, and UBAP1) in TSG101-depleted cells, likely due to destabilization of the ESCRT-Ⅰ complex by the TSG101 depletion (Fig. 2 C). HRS and STAM1/2, direct substrates of USP8, were downregulated by approximately half (Fig. 2 C and Table S3). A gene ontology (GO) biological process enrichment test using the DAVID platform showed that proteins annotated in the categories related to the immune system (e.g., defense response to virus, type Ⅰ interferon signaling pathway, negative regulation of viral genome regulation, and innate immune response) were enriched in proteins that were increased in USP8-depleted cells (Fig. 2, D and E). CCL5 (also known as RANTES) protein showed the largest increase in both USP8- and TSG101-depleted cells (Fig. 2, C and E). CCL5, a chemokine released by T cells, recruits leukocytes to the site of inflammation (Appay and Rowland-Jones, 2001). We confirmed the upregulation of CCL5 in USP8-depleted cells by immunoblotting (Fig. 2 F and Fig. S2 C). Thus, our proteomic screen identified a link between endosomal stress and activation of the immune response pathway.

### Endosomal stress activates TAK1–NF-κB signaling by recruiting TAB2/3

**Figure 3.**
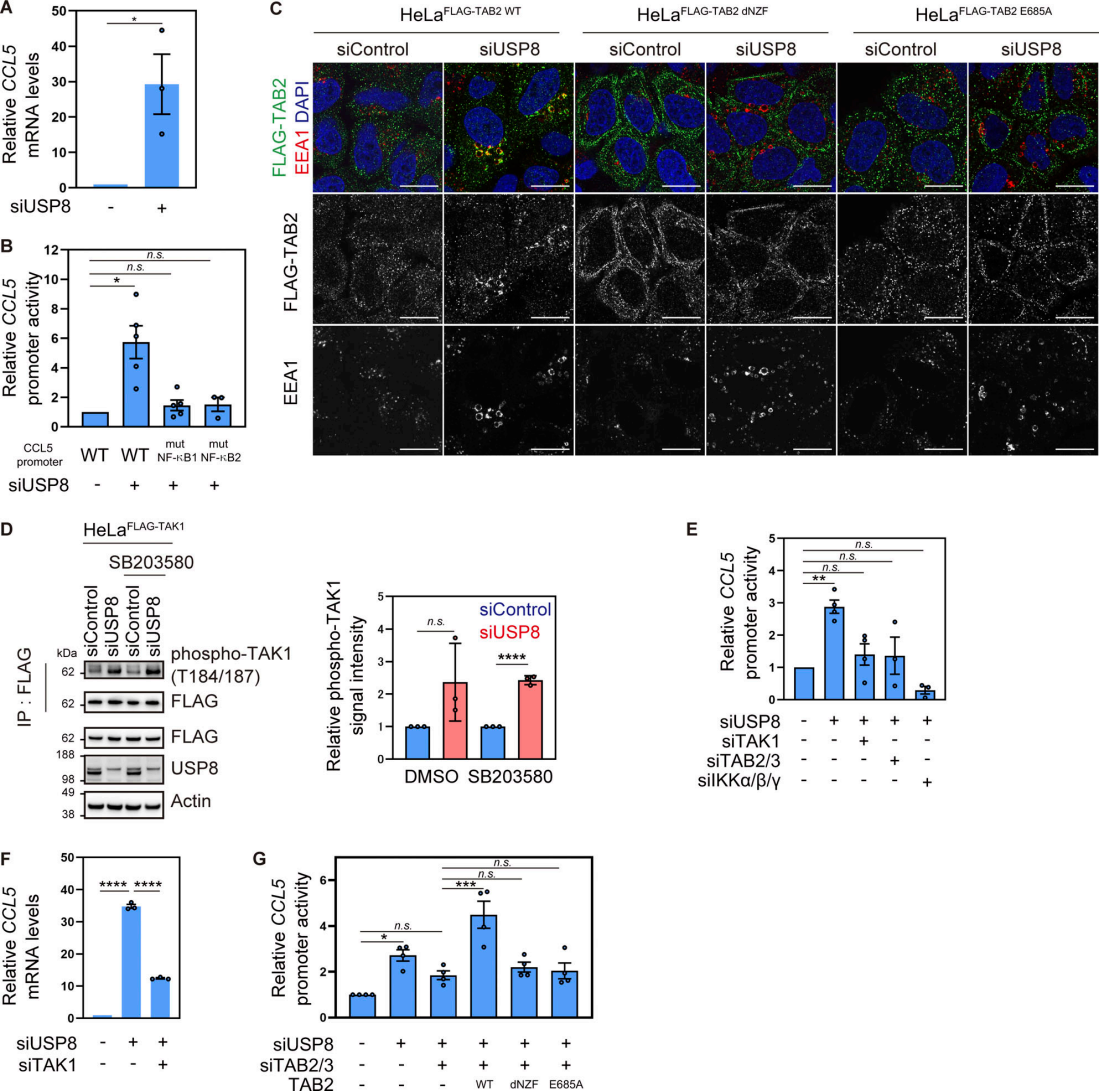
**Endosomal stress activates TAK1–NF-**κ**B signaling by recruiting TAB2/3. (A)** Total RNA from HeLa cells transfected with the indicated siRNAs was analyzed by qRT-PCR. CCL5 expression levels were normalized to actin mRNA levels, and expression levels in cells treated with control siRNA were set to 1. Individual values, mean, and SE of the mean of the relative mRNA levels are shown. Means ± SE were calculated from three biological replicates. *P < 0.05 (two-tailed Student’s *t* test). **(B)** HeLa cells transfected with the indicated siRNAs were analyzed by luciferase assay using vectors encoding either WT *CCL5* promoter or mutants (mut) lacking NF-κB binding sites. The activities of the *CCL5* promoter were normalized to those of the phosphoglycerate kinase (PGK) promoter, and the relative activities of the WT *CCL5* promoter in cells treated with control siRNA were set to 1. Individual values, mean, and SE of the mean of the relative promoter activities are shown. Means ± SE were calculated from three and five biological replicates. *P < 0.05 (Kruskal–Wallis and Dunn’s tests). **(C)** HeLa cells stably expressing FLAG-TAB2 WT, dNZF, and E685A were transfected with the indicated siRNAs. Cells were immunostained with the indicated antibodies and DAPI. Scale bar, 20 μm. **(D)** HeLa cells stably expressing FLAG-TAK1 were transfected with the indicated siRNAs and treated with or without SB203580. FLAG-TAK1 immunoprecipitated with anti-FLAG antibody and total cell lysates were immunoblotted with the indicated antibodies (left). Signal intensities of phospho-TAK1 were quantified and normalized to those of total immunoprecipitated FLAG-TAK1 (right). Relative intensities in cells treated with control siRNA were set to 1. Individual values, mean, and SD of the mean of relative intensities are shown. Means ± SD were calculated from three biological replicates. ****P < 0.0001 (two-tailed Student’s *t* test). **(E)** HeLa cells transfected with the indicated siRNAs were analyzed by luciferase assay using vectors encoding the WT *CCL5* promoter. The activities of the *CCL5* promoter were normalized to those of the PGK promoter, and the relative activities of the WT *CCL5* promoter in cells treated with control siRNA were set to 1. Individual values, mean, and SE of the mean of the relative promoter activities are shown. Means ± SE were calculated from three and four biological replicates. **P < 0.01 (one-way ANOVA with Dunnett’s test). **(F)** Total RNA from HeLa cells transfected with the indicated siRNAs was analyzed by qRT-PCR. CCL5 expression levels were normalized to actin mRNA levels, and expression levels in cells treated with control siRNA were set to 1. Individual values, mean, and SE of the mean of relative mRNA levels are shown. Means ± SE were calculated from three biological replicates. ****P < 0.0001 (one-way ANOVA with Dunnett’s test). **(G)** HeLa cells transfected with the indicated siRNAs and vectors to express the indicated proteins were analyzed by luciferase assay using vectors encoding the WT *CCL5* promoter. The activities of the *CCL5* promoter were normalized to those of the thymidine kinase (TK) promoter, and the relative activities of the WT *CCL5* promoter in cells treated with control siRNA were set to 1. Individual values, mean, and SE of the mean of the relative promoter activities are shown. Means ± SE were calculated from four biological replicates. *P < 0.05, ***P < 0.001 (one-way ANOVA with Dunnett’s test). Source data are available for this figure: SourceData F3.

**Figure S3.**
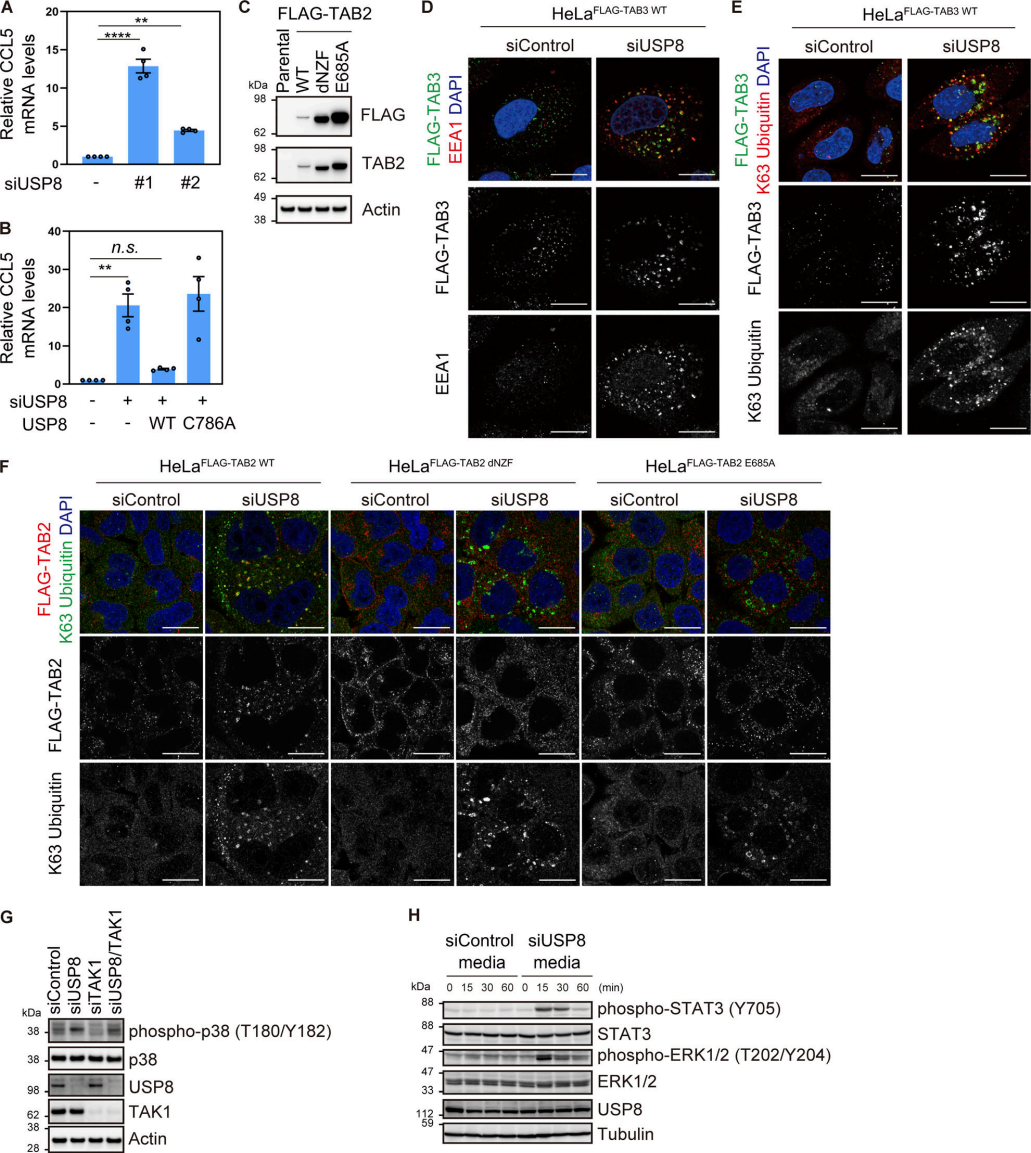
**TAB2/3-mediated signal transduction in USP8-depleted cells. (A)** Total RNA from HeLa cells transfected with the indicated siRNAs was analyzed by qRT-PCR. CCL5 expression levels were normalized to actin mRNA levels, and expression levels in cells treated with control siRNA were set to 1. Individual values, mean, and SE of the mean of relative mRNA levels are shown. Means ± SE were calculated from four biological replicates. **P < 0.01, ****P < 0.0001 (one-way ANOVA with Dunnett’s test). **(B)** Parental HeLa cells and HeLa cells stably expressing siRNA-resistant Myc-USP8 WT and C786A were transfected with the indicated siRNAs. Total RNA from cells was analyzed by qRT-PCR. CCL5 expression levels were normalized to actin mRNA levels, and expression levels in cells treated with control siRNA were set to 1. Individual values, mean, and SE of the mean of the relative mRNA levels are shown. Means ± SE were calculated from four biological replicates. **P < 0.01 (one-way ANOVA with Dunnett’s test). **(C)** Total cell lysates from parental HeLa cells and HeLa cells stably expressing FLAG-TAB2 WT, dNZF, and E685A were immunoblotted with the indicated antibodies. **(D and E)** HeLa cells stably expressing FLAG-TAB3 WT were transfected with the indicated siRNAs. Cells were immunostained with the indicated antibodies and DAPI. Scale bar, 20 μm. **(F)** HeLa cells stably expressing FLAG-TAB2 WT, dNZF, and E685A were transfected with the indicated siRNAs. Cells were immunostained with the indicated antibodies and DAPI. Scale bar, 20 μm. **(G)** Total cell lysates from HeLa cells transfected with the indicated siRNAs were immunoblotted with the indicated antibodies. **(H)** HeLa cells were stimulated with culture media from HeLa cells transfected with the indicated siRNAs. Total cell lysates were immunoblotted with the indicated antibodies. Source data are available for this figure: SourceData FS3.

To examine whether the upregulation of CCL5 protein in the USP8-depleted cells was due to the increase in mRNA levels, we performed quantitative RT-PCR (qRT-PCR) analysis and found that CCL5 mRNA was dramatically increased (Fig. 3 A and Fig. S3 A). The increase in CCL5 mRNA in USP8-depleted cells was rescued by the add-back of USP8 in a deubiquitinating activity-dependent manner (Fig. S3 B). We next examined *CCL5* promoter activity using the ∼1 kb upstream region of the human CCL5 gene (Fig. 3 B). The *CCL5* promoter has been shown to contain multiple transcriptional responsive elements, and gene activation upon various stimuli has been shown to involve two NF-κB binding sites (Fessele et al., 2002). *CCL5* WT promoter activity was increased by approximately sixfold in USP8-depleted cells, consistent with the increase in CCL5 mRNA levels (Fig. 3 B). However, the mutations in either of the two NF-κB elements abolished *CCL5* promoter activation induced by USP8 depletion (Fig. 3 B). These results suggest that the *CCL5* gene is activated in an NF-κB–dependent manner in USP8-depleted cells.

Since USP8 depletion causes the accumulation of K63 ubiquitin chains on endosomes, we hypothesized that the K63 ubiquitin signals directly activate NF-κB via ubiquitin decoders. In the NF-κB pathway, TAB2 and TAB3, known as a paralog of TAB2, function as ubiquitin decoders that specifically recognize K63 ubiquitin chains via the C-terminal RanBP2-like zinc finger (NZF) domain (Kanayama et al., 2004; Kulathu et al., 2009). We therefore generated cells stably expressing FLAG-tagged TAB2 and TAB3, as well as TAB2 mutants lacking ubiquitin-binding ability, dNZF and E685A (substitution of Glu 685, the critical amino acid for ubiquitin binding in the NZF domain, with Ala; Fig. S3 C) and investigated their subcellular localization in USP8-depleted cells. We observed translocation of stably expressed TAB2 and TAB3 adjacent to K63 ubiquitin chains on early endosomes (Fig. 3 C and Fig. S3, D–F). In contrast, TAB2 dNZF and E685A mutants did not colocalize with K63 ubiquitin chains on endosomes upon USP8 depletion (Fig. 3 C and Fig. S3 F). These demonstrate that TAB2 is recruited to endosomes by binding to accumulated K63 ubiquitin chains via the NZF domain.

Association of TAB2/3 with K63 ubiquitin chains has been shown to activate TAK1 in cells stimulated with cytokines such as TNF-α and IL-1 (Kanayama et al., 2004; Wang et al., 2001). Since unanchored K63 ubiquitin chains can sufficiently activate TAK1 in vitro (Xia et al., 2009), K63 ubiquitin chains on endosomes would facilitate TAB2/3-dependent activation of TAK1. To monitor the kinase activity of TAK1 upon USP8 depletion, stably expressed FLAG-TAK1 was affinity-purified with anti-FLAG antibody and analyzed by immunoblotting with anti-phosphorylated TAK1 (a major active form) antibody (Fig. 3 D). We treated cells with the p38 MAPK inhibitor SB203580 because p38 MAPK is a downstream substrate of TAK1 and also acts as a negative regulator of TAK1 phosphorylation (Moriguchi et al., 1996; Strippoli et al., 2010). The phosphorylation levels of FLAG-TAK1 were not significantly affected in USP8-depleted cells but increased in the presence of SB203580, suggesting that TAK1 is activated upon USP8 depletion and undergoes negative feedback regulation by p38 MAPK. In support of this, phosphorylation levels of p38 MAPK were increased upon USP8 depletion, whereas additional depletion of TAK1 with USP8 partially abolished p38 MAPK phosphorylation (Fig. S3 G). Activated TAK1 phosphorylates downstream targets such as subunits of the IKK complex, IKKα, β, and γ (Kanayama et al., 2004; Wang et al., 2001), resulting in NF-κB–mediated gene expression. Luciferase assays using the *CCL5* WT promoter showed that additional depletion of either TAK1, TAB2/3, or all the IKK subunits (α, β, and γ) with USP8 at least partially abolished *CCL5* promoter activation (Fig. 3 E). Consistently, CCL5 mRNA levels increased upon USP8 depletion were suppressed by additional depletion of TAK1 (Fig. 3 F). Importantly, the add-back of TAB2 WT, but not dNZF and E685A mutants, which are not recruited to endosomes, rescued *CCL5* promoter activation in USP8- and TAB2/3-depleted cells (Fig. 3 G). These indicate that TAB2 recruitment to endosomes and downstream NF-κB activation upon endosomal stress are mediated by NZF-dependent interaction with K63 ubiquitin chains.

Since chemokines, including CCL5, function as intercellular signal transducers after secretion into the extracellular space (Luther and Cyster, 2001), we investigated whether biomolecules secreted from USP8-depleted cells sufficiently induce intercellular signal transduction. In HeLa cells treated with media collected from USP8-depleted cells, the phosphorylation levels of STAT3 and MAPK ERK1/2, which are key cellular signaling mediators, were dramatically increased 15 min after media replacement (Fig. S3 H).

Collectively, these data suggest that endosomal stress recruits TAB2/3 to endosomes via K63 ubiquitin chains and activates the TAK1–NF-κB pathway, leading to the expression of immune-related genes and intercellular signal transduction.

### Endosomal stress activates p62–Keap1–Nrf2 signaling

**Figure 4.**
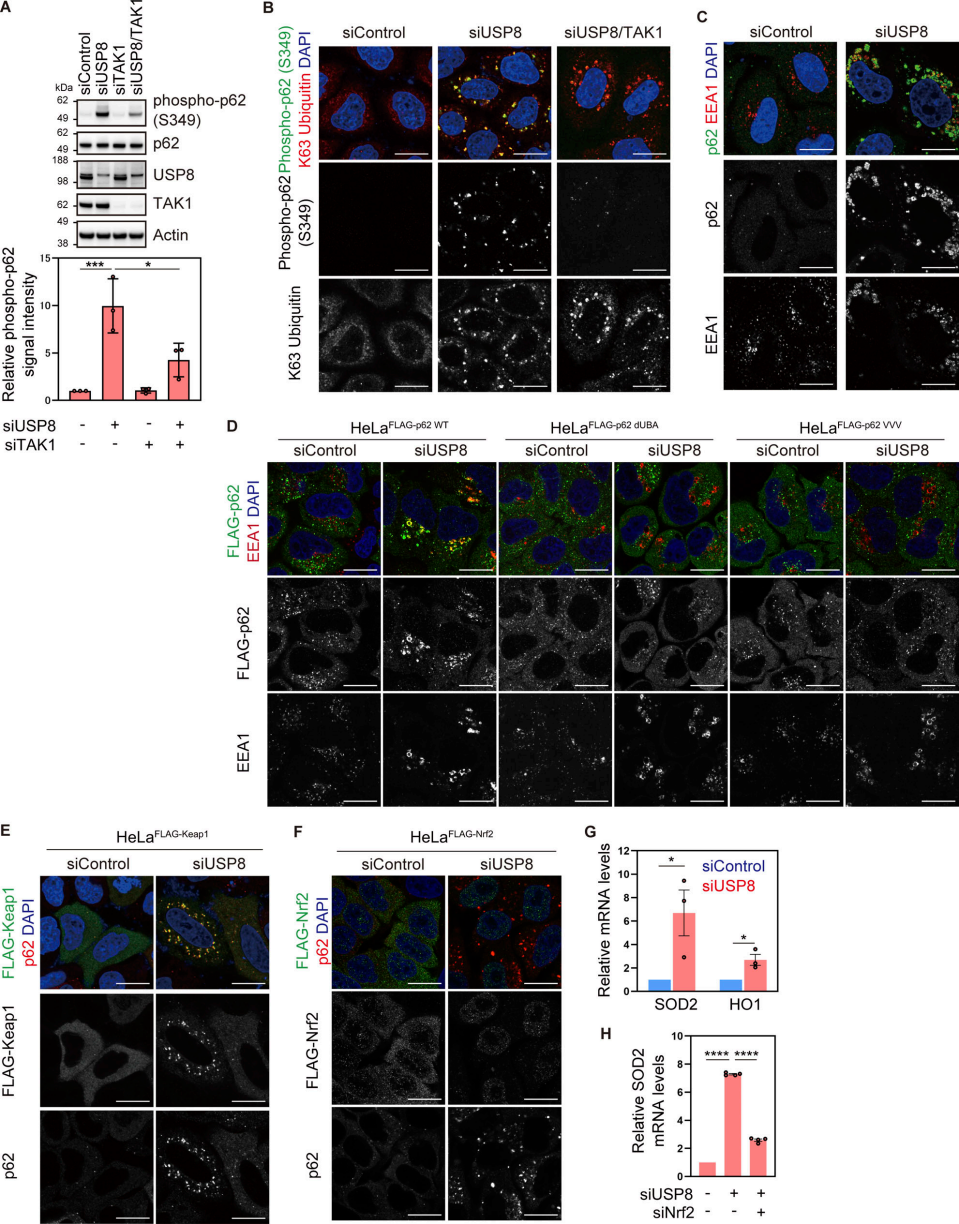
**Endosomal stress activates p62–Keap1–Nrf2 signaling. (A)** Total cell lysates from HeLa cells transfected with the indicated siRNAs were immunoblotted with the indicated antibodies (top). Signal intensities of phospho-p62 were quantified and normalized to those of total p62 (bottom). Relative intensities in cells treated with control siRNA were set to 1. Individual values, mean, and SD of the mean of relative intensities are shown. Means ± SD were calculated from three biological replicates. *P < 0.05, ***P < 0.001 (one-way ANOVA with Dunnett’s test). **(B and C)** HeLa cells transfected with the indicated siRNAs were immunostained with the indicated antibodies and DAPI. Scale bar, 20 μm. **(D)** HeLa cells stably expressing FLAG-p62 WT, dUBA, and VVV were transfected with the indicated siRNAs. Cells were immunostained with the indicated antibodies and DAPI. Scale bar, 20 μm. **(E)** HeLa cells stably expressing FLAG-Keap1 were transfected with the indicated siRNAs. Cells were immunostained with the indicated antibodies and DAPI. Scale bar, 20 μm. **(F)** HeLa cells stably expressing FLAG-Nrf2 were transfected with the indicated siRNAs. Cells were immunostained with the indicated antibodies and DAPI. Scale bar, 20 μm. **(G)** Total RNA from HeLa cells transfected with the indicated siRNAs was analyzed by qRT-PCR. Expression levels of SOD2 and HO1 were normalized to actin mRNA levels, and expression levels in cells treated with control siRNA were set to 1. Individual values, mean, and SE of the mean of relative mRNA levels are shown. Means ± SE were calculated from three biological replicates. *P < 0.05 (two-tailed Student’s *t* test). **(H)** Total RNA from HeLa cells transfected with the indicated siRNAs was analyzed by qRT-PCR. SOD2 expression levels were normalized to actin mRNA levels, and expression levels in cells treated with control siRNA were set to 1. Individual values, mean, and SE of the mean of the relative mRNA levels are shown. Means ± SE were calculated from four biological replicates. ****P < 0.0001 (one-way ANOVA with Dunnett’s test). Source data are available for this figure: SourceData F4.

**Figure S4.**
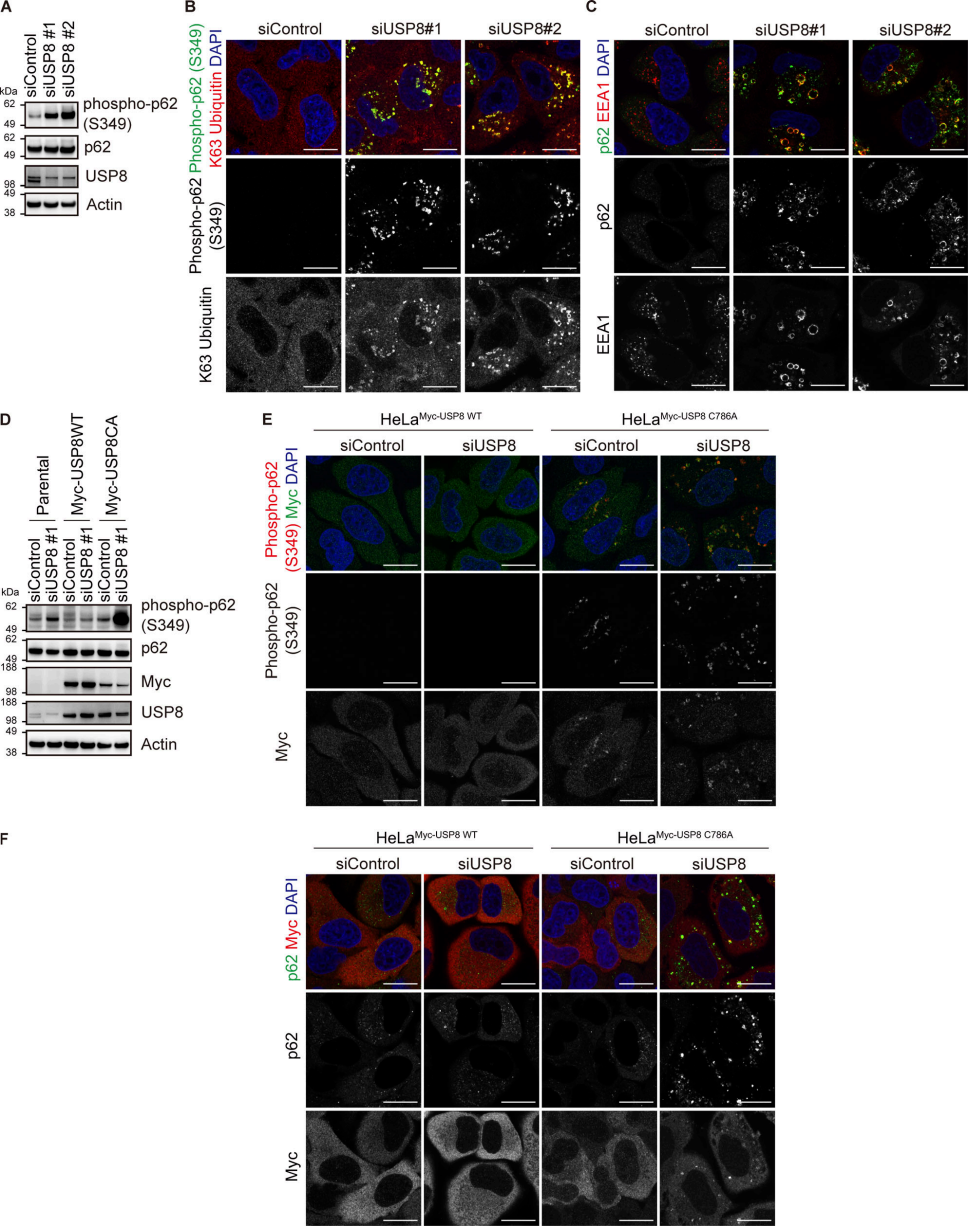
**Accumulation of phosphorylated p62 on endosomes in USP8-depleted cells. (A)** Total cell lysates from HeLa cells transfected with the indicated siRNAs were immunoblotted with the indicated antibodies. **(B and C)** HeLa cells transfected with the indicated siRNAs were immunostained with the indicated antibodies and DAPI. Scale bar, 20 μm. **(D)** Parental HeLa cells and HeLa cells stably expressing siRNA-resistant Myc-USP8 WT and C786A were transfected with the indicated siRNAs. Total cell lysates from cells were immunoblotted with the indicated antibodies. **(E and F)** HeLa cells stably expressing siRNA-resistant Myc-USP8 WT and C786A were transfected with the indicated siRNAs. Cells were immunostained with the indicated antibodies and DAPI. Scale bar, 20 μm. Source data are available for this figure: SourceData FS4.

**Figure S5.**
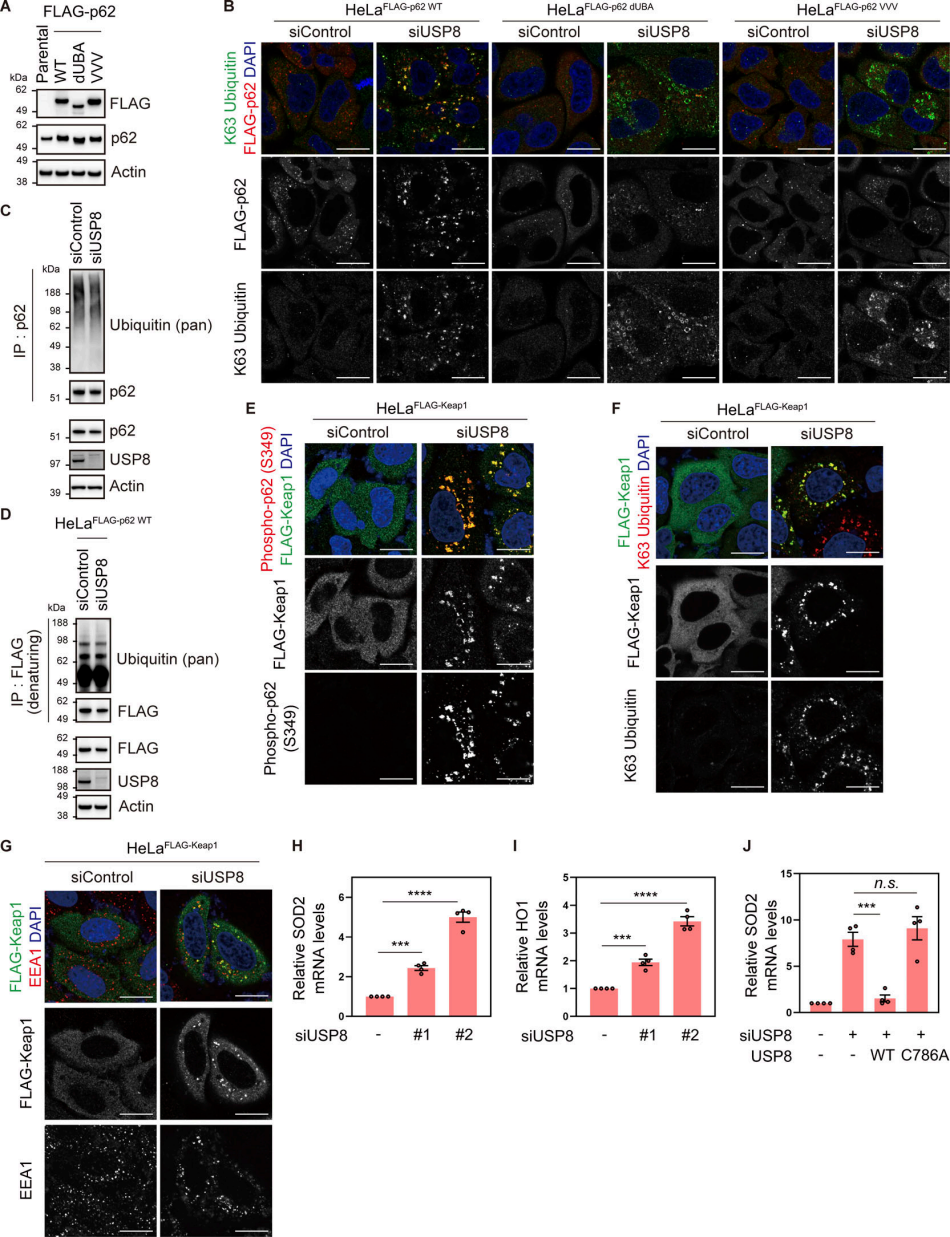
**Colocalization of p62 and Keap1 on endosomes in USP8-depleted cells. (A)** Total cell lysates from parental HeLa cells and HeLa cells stably expressing FLAG-p62 WT, dUBA, and VVV were immunoblotted with the indicated antibodies. **(B)** HeLa cells stably expressing FLAG-p62 WT, dUBA, and VVV were transfected with the indicated siRNAs. Cells were immunostained with the indicated antibodies and DAPI. Scale bar, 20 μm. **(C)** HeLa cells were transfected with the indicated siRNAs. Endogenous p62 immunoprecipitated with anti-p62 antibody and total cell lysates were immunoblotted with the indicated antibodies. **(D)** HeLa cells stably expressing FLAG-p62 WT were transfected with the indicated siRNAs. FLAG-p62 immunoprecipitated with anti-FLAG antibody under denaturing conditions and total cell lysates were immunoblotted with the indicated antibodies. **(E–G)** HeLa cells stably expressing FLAG-Keap1 were transfected with the indicated siRNAs. Cells were immunostained with the indicated antibodies and DAPI. Scale bar, 20 μm. **(H and I)** Total RNA from HeLa cells transfected with the indicated siRNAs was analyzed by qRT-PCR. SOD2 (H) and HO1 (I) expression levels were normalized to actin mRNA levels, and expression levels in cells treated with control siRNA were set to 1. Individual values, mean, and SE of the mean of relative mRNA levels are shown. Means ± SE were calculated from four biological replicates. ***P < 0.001, ****P < 0.0001 (one-way ANOVA with Dunnett’s test). **(J)** Parental HeLa cells and HeLa cells stably expressing siRNA-resistant Myc-USP8 WT and C786A were transfected with the indicated siRNAs. Total RNA from cells was analyzed by qRT-PCR. SOD2 expression levels were normalized to actin mRNA levels, and expression levels in cells treated with control siRNA were set to 1. Individual values, mean, and SE of the mean of relative mRNA levels are shown. Means ± SE were calculated from four biological replicates. ***P < 0.001 (one-way ANOVA with Dunnett’s test). Source data are available for this figure: SourceData FS5.

p62/Sequestosome-1, a multifunctional ubiquitin decoder, harbors ubiquitin-associated (UBA) domains in the C-terminal region that are critical for ubiquitin recognition (Sánchez-Martín and Komatsu, 2018). Numerous studies have demonstrated important roles for p62 in ubiquitin-mediated signaling, particularly in the autophagy machinery (Moscat et al., 2016). TAK1 has been reported to phosphorylate S349 of p62 (Hashimoto et al., 2016). Therefore, we investigated whether another ubiquitin decoder, p62, is affected downstream of USP8 depletion. Phosphorylated p62 at S349 was dramatically increased and colocalized with K63 ubiquitin chains, and a part of p62 was recruited to early endosomes in USP8-depleted cells (Fig. 4, A–C; and Fig. S4, A–C). The increase in p62 phosphorylation levels, the colocalization of phosphorylated p62 with K63 ubiquitin chains, and the accumulation of p62 on endosomes in USP8-depleted cells were abolished by the add-back of USP8 in a deubiquitinating activity-dependent manner (Fig. S4, D–F). We then generated cells stably expressing FLAG-tagged p62 as well as mutants lacking ubiquitin-binding ability, dUBA and VVV (substitution of ^404^MGF^406^ in the UBA domain with VVV; Hocking et al., 2004; Seibenhener et al., 2004; Fig. S5 A), and investigated their subcellular localization in USP8-depleted cells. p62 WT, but not dUBA and VVV mutants, colocalized with K63 ubiquitin chains on endosomes (Fig. 4 D and Fig. S5 B). This suggests that p62 is also recruited to endosomes by binding to accumulated K63 ubiquitin chains through the UBA domain. It has been reported that USP8 deubiquitinates p62 (Peng et al., 2020), but USP8 depletion had little effect on the total ubiquitination levels of p62 under our experimental conditions (Fig. S5, C and D).

p62 interacts with Keap1, an oxidative stress sensor E3 ligase, via a Keap1-interacting region adjacent to a UBA domain in a phosphorylated S349-dependent manner (Ichimura et al., 2013; Komatsu et al., 2010). In USP8-depleted cells, stably expressed FLAG-Keap1 colocalized with p62, EEA1, K63 ubiquitin chains, and phosphorylated p62 at S349 (Fig. 4 E and Fig. S5, E–G). Keap1 constitutively ubiquitinates and destabilizes Nrf2, a master transcription factor of the stress response, through proteasomal degradation (Itoh et al., 1999). Under oxidative stress, oxidation occurs on several cysteines of Keap1, causing conformational changes in the Keap1 structure and reducing the affinity between Keap1 and Nrf2 (Itoh et al., 1999). In addition to the oxidation-dependent Keap1 inactivation, there is p62-mediated Nrf2 activation in which p62 phosphorylation at S349 acquires high affinity for Keap1 and competes with constitutive Keap1–Nrf2 binding (Ichimura et al., 2013). In both cases, free Nrf2 released from Keap1 is translocated to the nucleus and induces the expression of target genes such as *SOD2* and *HO1*, which counteract the cellular oxidative environment and contribute to cell survival (Mohammadzadeh et al., 2012). Consistent with these previous reports, we found that stably expressed FLAG-Nrf2 was translocated to the nuclei and that the mRNA levels of SOD2 and HO1 were increased in USP8-depleted cells (Fig. 4, F and G; and Fig. S5, H and I). The increase in SOD2 mRNA in USP8-depleted cells was rescued by the add-back of USP8 in a deubiquitinating activity-dependent manner (Fig. S5 J). Furthermore, the increase in SOD2 mRNA levels upon USP8 depletion was suppressed by additional depletion of Nrf2 (Fig. 4 H). These results suggest that p62 associates with K63 ubiquitin chains on endosomes and undergoes TAK1-mediated phosphorylation upon endosomal stress, leading to nuclear translocation and activation of Nrf2 through sequestration of Keap1.

### Oxidative stress is a potent stimulus to induce endosomal stress

**Figure 5.**
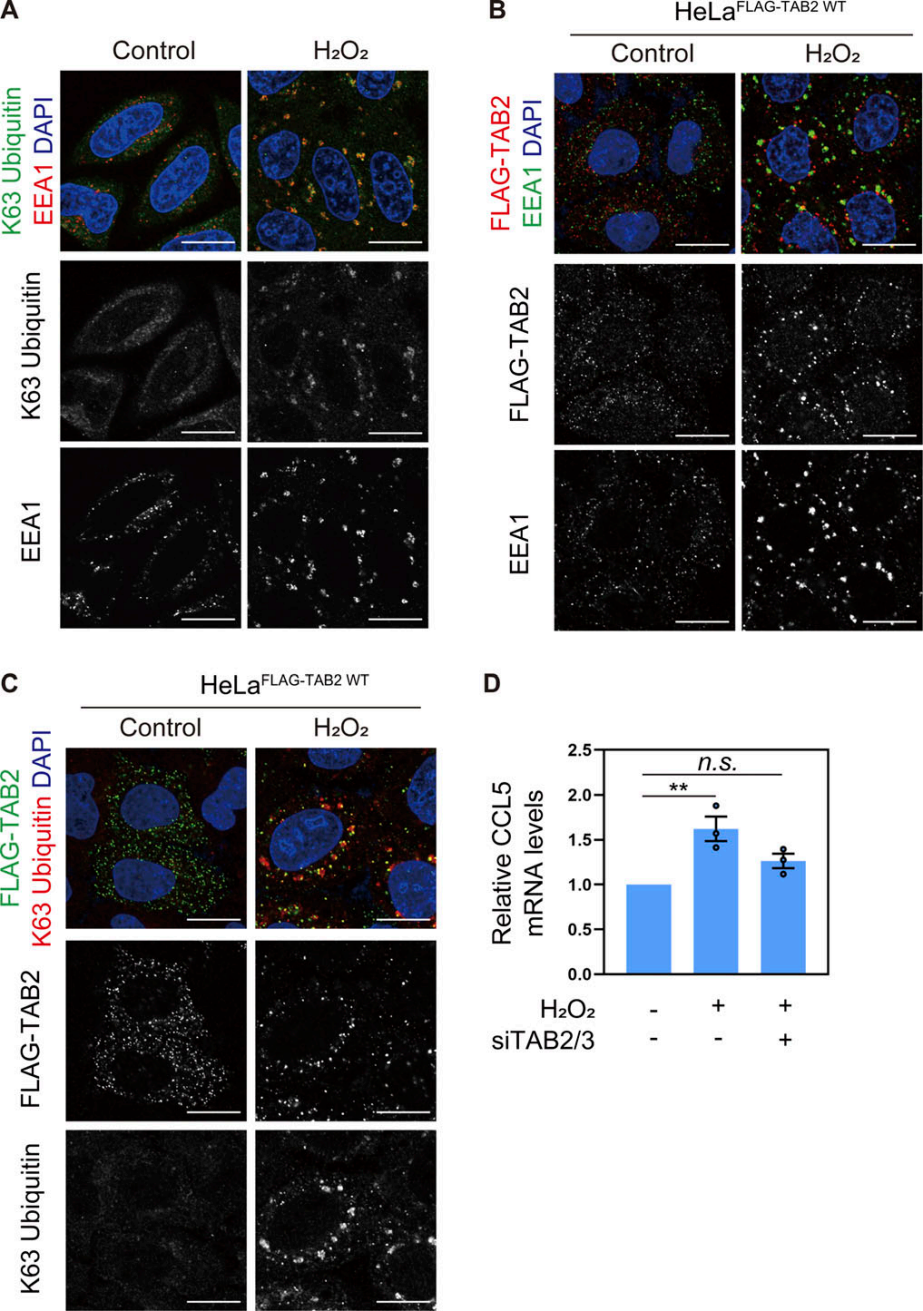
**Oxidative stress is a potent stimulus to induce endosomal stress. (A)** HeLa cells treated with 300 μM hydrogen peroxide (H_2_O_2_) for 4 h were immunostained with the indicated antibodies and DAPI. Scale bar, 20 μm. **(B and C)** HeLa cells stably expressing FLAG-TAB2 WT were treated with 300 μM hydrogen peroxide for 4 h. Cells were immunostained with the indicated antibodies and DAPI. Scale bar, 20 μm. **(D)** Total RNA from HeLa cells transfected with the indicated siRNAs and treated with 300 μM hydrogen peroxide for 4 h was analyzed by qRT-PCR. CCL5 expression levels were normalized to actin mRNA levels, and expression levels in cells treated with control siRNA were set to 1. Individual values, mean, and SE of the mean of relative mRNA levels are shown. Means ± SE were calculated from three biological replicates. **P < 0.01 (one-way ANOVA with Dunnett’s test).

Finally, we sought environmental stimuli that could induce endosomal stress and investigated whether hydrogen peroxide, which has been reported to decrease the enzymatic activity of USP8 by oxidizing catalytic cysteine in vitro, induces endosomal stress (Lee et al., 2013). In Hela cells treated with 300 µM H_2_O_2_ for 4 h, we observed enlarged endosomes and accumulation of K63 ubiquitin chains on endosomes (Fig. 5 A). Under these conditions, stably expressed FLAG-TAB2 was recruited to K63 ubiquitin chains accumulated on endosomes and the CCL5 mRNA level was increased in a TAB2/3-mediated manner (Fig. 5, B–D). These results suggest that oxidative stress is an environmental stimulus to induce endosomal stress.

## Discussion

**Figure 6.**
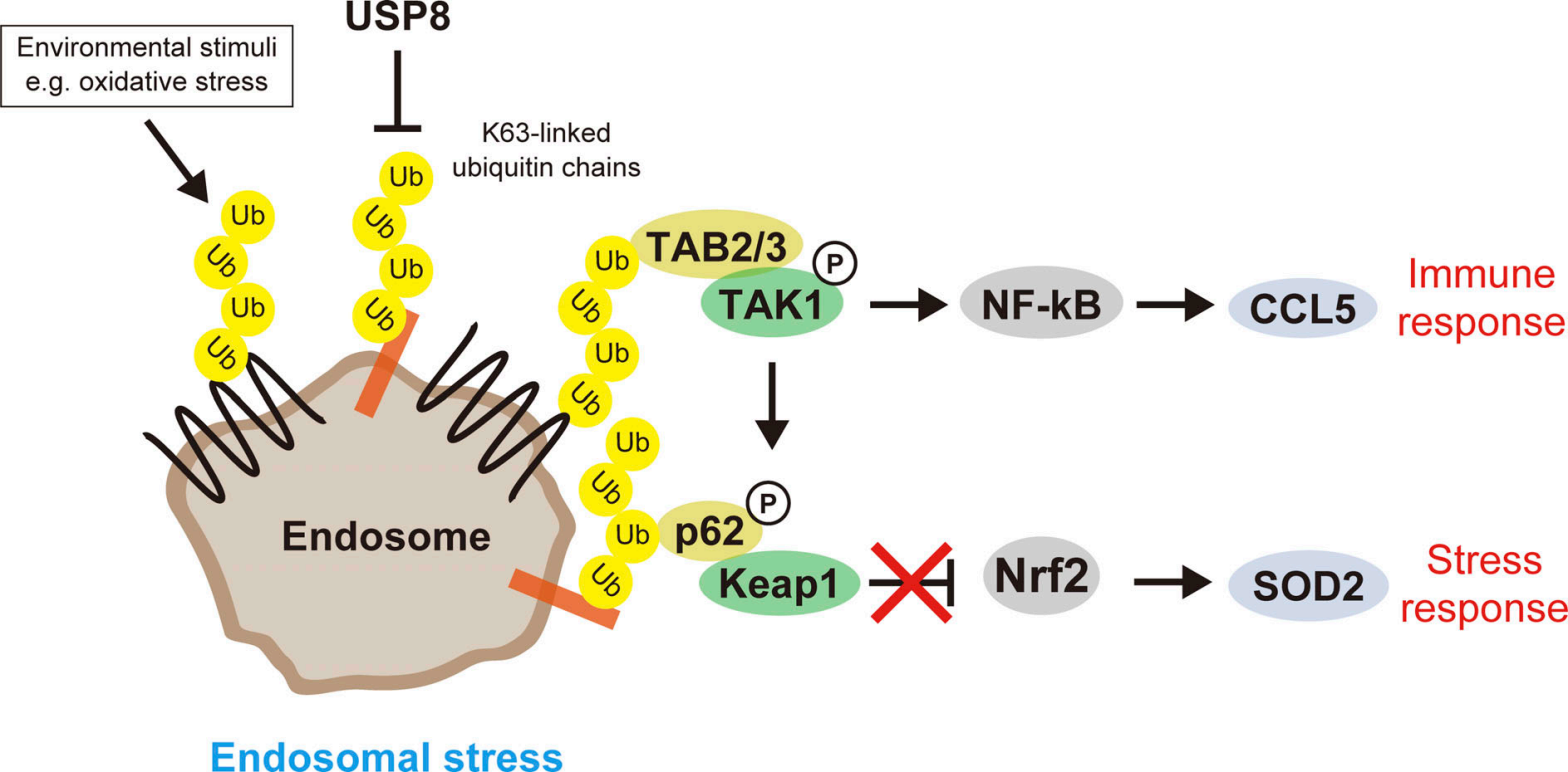
Model of endosomal stress.

In this study, we termed the aberrant accumulation of K63 ubiquitin chains on functionally defective endosomes in USP8-depleted cells endosomal stress. Endosomal stress activated TAB2/3–TAK1–NF-κB and p62–Keap1–Nrf2 pathways, resulting in the expression of target genes such as *CCL5* and *SOD2*. Upon endosomal stress, TAB2/3 and p62 decode excess K63 ubiquitin chains accumulated on endosomes and transduce immune and stress response signals from endosomes. In addition to RNAi-mediated USP8 depletion, signatures of endosomal stress (i.e., K63 ubiquitin accumulation on enlarged endosomes accompanied by TAB2 recruitment) were observed in cells exposed to oxidative stress, which can inactivate USP8. Therefore, we hypothesized that USP8 is a gatekeeper of endosomal stress (Fig. 6).

In mice, conventional knockout of USP8 causes embryonic lethality early in development and conditional knockout leads to rapid death in adult mice (Niendorf et al., 2007). In addition, T cell–specific conditional knockout of USP8 has been reported to abolish T cell maturation and the immune system (Dufner et al., 2015). Together with the clinical report that mutations in the USP8 gene cause Cushing’s disease, these findings support the critical roles of USP8 in cellular functions. However, the molecular mechanisms by which loss of USP8 leads to these phenotypes have not been fully understood. In this study, we showed that USP8 is a potent upstream repressor of NF-κB and Nrf2, master transcription factors of immune and stress responses, respectively, by preventing endosomal stress. In agreement with this, Xiong and colleagues recently reported that USP8 inhibition triggers the innate immune response by activating the NF-κB pathway in CT26 cells (murine colorectal carcinoma; Xiong et al., 2022). Importantly, our proteome and their transcriptome analyses are consistent with the observation that USP8 depletion activates the immune response, suggesting that USP8-mediated regulation of the immune response is ubiquitous across cell types. In addition, we showed that biomolecules secreted from cells under endosomal stress sufficiently stimulated signal transduction in neighboring cells (Fig. S3 H). This implies that such endosomal stress–mediated signal transduction occurs in vivo, although it remains to be determined which tissues or cell types are vulnerable to endosomal stress. Recently, it has been reported that USP8 expression is downregulated in hepatic macrophages of patients with cirrhosis (Moreno-Lanceta et al., 2023), possibly implicating the link between aberrant signaling caused by endosomal stress and pathology. Hydrogen peroxide–induced oxidative stress, which inhibits the activity of USP8, caused endosomal stress, suggesting that physiological and pathological conditions associated with impairment of USP8 function induce endosomal stress. It should be acknowledged that oxidation inhibits not only USP8 but also a wide range of enzymes, and therefore we would like to seek physiological stimuli that more specifically reduce the activity of USP8 in the future.

K63 ubiquitin chains are involved in diverse cellular functions throughout the cell. Endocytosis and endosome-to-lysosome sorting have been well characterized as mediated by K63 ubiquitin chains. To date, multiple decoders for K63 ubiquitin chains have been identified, but the spatiotemporal mechanism by which they are utilized for specific downstream consequences remains poorly understood. We found that TAB2/3 and p62, major decoders for K63 ubiquitin chains, recognize ubiquitinated substrates on endosomes upon endosomal stress caused by USP8 depletion and trigger signaling pathways from endosomes. Presumably, TAB2/3 and p62 recognize K63 ubiquitin chains, originally formed as tags for endocytosis and lysosomal sorting and retained on defective endosomes upon USP8 depletion. In this context, our findings may exemplify redecoding of K63 ubiquitin chains from membrane trafficking to signal transduction by switching decoder proteins.

## Materials and methods

### Cell culture

HeLa cells were grown at 37°C under 5% CO_2_ in Dulbecco’s Modified Eagle Medium (DMEM; Thermo Fisher Scientific) supplemented with 10% fetal bovine serum (FBS; Biowest), 100 U/ml penicillin, and 100 µg/ml streptomycin (Thermo Fisher Scientific). Cells were grown in DMEM supplemented with 0.5% FBS 16 h before harvest. Cells were stimulated with culture media from siRNA-transfected cells for the indicated times. Cells were treated with 20 μM SB203580 (Adipogen Life Science) and 300 µM hydrogen peroxide (H_2_O_2;_ WAKO) for the indicated time.

### siRNA transfection

Cells were transfected with siRNAs using Lipofectamine RNAiMax (Thermo Fisher Scientific) at a final siRNA concentration of 30 nM according to the manufacturer’s protocol. Cells were harvested and analyzed 48 or 72 h after siRNA transfection. siRNAs utilized pools of four different sequences (Thermo Fisher Scientific). USP8 siRNAs: L-005203, TSG101 siRNAs: L-003549, TAK1 siRNAs: L-003790, TAB2 siRNAs: L-004771, TAB3 siRNAs: L-015572, IKKα siRNAs: L-003473, IKKβ siRNAs: L-003503, IKKγ siRNAs: L-003763, and Nrf2 siRNAs: L-003755. For USP8 individual siRNAs, J-005203-06 and -07 were used as #1 and #2, respectively.

### Absolute quantification of ubiquitin linkages (Ub-AQUA/PRM)

MS/MS-based Ub-AQUA/PRM was performed as previously described (Tsuchiya et al., 2013). Briefly, for total cell lysate (10 µg) and membrane fractions (5 µg) obtained using a subcellular fractionation kit (Thermo Fisher Scientific), proteins were separated on NuPAGE gels (Thermo Fisher Scientific) with a short run (1 cm). After in-gel trypsin digestion, extracted peptides were spiked with Ub-AQUA peptides (K48 and K63 linkages), concentrated using a speed-vac, and then oxidized with 0.05% H_2_O_2_/0.1% trifluoroacetic acid (TFA) for 12 h. Peptides were analyzed by LC-MS/MS on an EASY-nLC 1200-connected Orbitrap Fusion Lumos Tribrid mass spectrometer (Thermo Fisher Scientific). Peptides were separated on an analytical column (C18, 1.6 µm particle size × 75 µm diameter × 250 mm; Ion Opticks) using a 45-min gradient (0–40% acetonitrile over 45 min) at a constant flow rate of 300 nl/min. Peptide ionization was performed using a Nanospray Flex Ion Source (Thermo Fisher Scientific). MS raw files were analyzed using PinPoint software 1.3 (Thermo Fisher Scientific).

### Immunofluorescence and confocal microscopy analysis

Cells were fixed with 4% paraformaldehyde in phosphate-buffered saline (PBS) for 10 min at room temperature (RT) and permeabilized with 0.2% Triton X-100 in PBS for 5 min at RT, or incubated with ice-cold methanol for 10 min on ice, followed by incubation with 5% FBS and 0.1% Tween in PBS for 1 h on ice. After blocking, cells were incubated with primary antibodies for 2 h at RT and then stained with secondary antibodies and 4′,6-diamidino-2-phenylindole (DAPI; Sigma-Aldrich) for 1 h at RT. Images were captured using ZEN 2012 and ZEN 3.8 imaging software and LSM780 and LSM980 laser-scanning confocal microscopes equipped with a Plan-Apochromat 63×/1.4NA oil lens (Carl Zeiss). The primary antibodies used for immunofluorescence were anti-K63 ubiquitin rabbit monoclonal (05-1308; clone Apu3; Millipore), anti-EEA1 mouse monoclonal (610457; clone 14; BD), anti-FLAG rabbit polyclonal (F7425; Sigma-Aldrich), anti-phospho-p62 (S349) mouse monoclonal (M217-3; clone 5D5; MBL), anti-p62 rabbit polyclonal (PM045; MBL), anti-DYKDDDDK tag mouse monoclonal (014-22383; clone 1E6; WAKO), anti-multi ubiquitin mouse monoclonal (D058-3; clone FK2; MBL), anti-Myc mouse monoclonal (clone 9E10), anti-LAMP1 rabbit monoclonal (9091; clone D2D11; Cell Signaling Technology), and anti-Myc rabbit polyclonal (562; MBL) antibodies. The secondary antibodies were purchased from Thermo Fisher Scientific: Alexa Fluor 488–conjugated anti-mouse and anti-rabbit, Alexa Fluor 594–conjugated anti-mouse, and anti-rabbit antibodies.

### Expression constructs, lentiviral transduction, and generation of stable HeLa cell lines

Human cDNAs for TAB2, TAB3, TAK1, Keap1, Nrf2, and USP8 were amplified from total mRNA of HEK293 cells, and the FLAG-Ub expression vector was kindly provided by Dr. T. Suzuki (Tokyo Metropolitan Institute of Medical Science). To generate a catalytically inactive mutant of USP8, a point mutation substituting Cys786 for Ala (C786A) was introduced by inverse PCR. The coding sequences for ubiquitin, TAB2, TAB3, TAK1, Keap1, Nrf2, USP8 WT, and USP8 C786A were cloned into pLVsin vector (Clontech). For lentivirus production, HEK293T cells were triple-transfected with two plasmids encoding lentivirus essential genes and either pLVsin-FLAG-Ub, -FLAG-TAB2, -FLAG-TAB3, -FLAG-TAK1, -FLAG-Keap1, -FLAG-Nrf2, -Myc-USP8 WT, or -Myc-USP8 C786A using polyethylenimine (linear; molecular weight, 25,000; Polyscience). 16 h after transfection, the medium was changed. 72 h after transfection, supernatants containing lentiviruses were filtered and concentrated. Lentiviruses were used to transduce cells in the presence of 8 µg/ml polybrene (Millipore). After transduction and appropriate antibiotic selection, each pool population was used for experiments. To generate siRNA-resistant FLAG-TAB2 WT, dNZF, E685A, p62 WT, dUBA, and VVV, their genes were synthesized with human-optimized codons. pZDONOR plasmids (Sigma-Aldrich) encoding them were knocked into the AAVS1 locus of HeLa cells using the TALEN kit, a gift from F. Zhang (1000000019; Addgene), with TALEN-targeting sequences AAVS1-1 (5′-TGT​CCC​CTC​CAC​CCC​ACA-3′) and AAVS1-2 (5′-TTT​CTG​TCA​CCA​ATC​CTG-3′; Sanjana et al., 2012).

### Immunoprecipitation (IP)

Cells were lysed in lysis buffer (20 mM Tris-HCl, pH 7.5, 100 mM NaCl, 50 mM NaF, 0.5% NP-40, 1 mM EDTA, 100 mM N-ethylmaleimide [NEM]) containing a complete protease inhibitor cocktail (EDTA-free; Roche) and a phosphatase inhibitor cocktail (Roche). For denaturing IP, cells were lysed in 1% SDS/radioimmunoprecipitation assay [RIPA] buffer (25 mM Tris-HCl, pH 8.0, 150 mM NaCl, 1% NP-40, 0.5% sodium deoxycholate, 100 mM NEM) containing a complete protease inhibitor cocktail (EDTA-free; Roche) and a phosphatase inhibitor cocktail (Roche). SDS was diluted to 0.1% prior to IP reactions. Anti-DYKDDDDK-tag mAb-agarose (M185-11; MBL) and anti-p62 rabbit polyclonal (PM045; MBL) were used to precipitate the target protein complex by incubation for 2 h at 4°C. After three washes with lysis buffer or 0.1% SDS/RIPA buffer, coimmunoprecipitates were subjected to downstream analyses.

### IP-MS analysis

Coimmunoprecipitates were subjected to trypsin digestion in solution. Briefly, co-immunoprecipitates eluted with 200 ng/μl FLAG peptide (F3290; Sigma-Aldrich) were reduced with 10 mM dithiothreitol (Thermo Fisher Scientific) and alkylated with 10 mM methyl methanethiosulfonate (Tokyo Chemical Industry). Proteins were precipitated with acetone and dried with a speed-vac. Proteins were resuspended in a solution of 8 M urea, 100 mM Tris-HCl, pH 8.0, and 1 mM CaCl_2_ and diluted to 0.8 M urea after complete solubilization. Trypsin (Thermo Fisher Scientific) was added at a final concentration of 0.4 ng/μl and samples were incubated for 16 h at 37°C. The resulting peptides were desalted using a tC18 96-well plate (Waters). Peptides were then resuspended in 0.1% TFA and analyzed by LC-MS/MS on an EASY-nLC 1200-connected Orbitrap Fusion Lumos Tribrid mass spectrometer (Thermo Fisher Scientific). Peptides were separated on an analytical column (C18, 1.6 µm particle size × 75 µm diameter × 250 mm; Ion Opticks) using a 120-min gradient (0–40% acetonitrile over 120 min) at a constant flow rate of 300 nl/min. Peptide ionization was performed using a Nanospray Flex Ion Source (Thermo Fisher Scientific). A “3-s cycle” data-dependent acquisition method was used, in which the most intense ions were selected every 3 s for MS/MS fragmentation by higher-energy collisional dissociation. MS raw files were analyzed using a Sequest HT search program in Proteome Discoverer 2.4 (Thermo Fisher Scientific). MS/MS spectra were searched against the SwissProt-reviewed human reference proteome (UniProt). Intensity-based non-label protein quantification was performed using a Precursor Ions Quantifier node in Proteome Discoverer 2.4.

### MS data processing and visualization

After processing with a Proteome Discoverer 2.4, the exported data were subjected to GO enrichment tests using DAVID (https://david.ncifcrf.gov/; Huang et al., 2009; Sherman et al., 2022) and aligned on the heatmaps using Morpheus (https://software.broadinstitute.org/morpheus/). Based on the generated data, the scatter plots and bubble plots were visualized using GraphPad Prism 8 (GraphPad).

### Immunoblotting

Cells were lysed in a solution of 2% SDS, 20 mM HEPES, pH 7.4, and 1 mM EDTA with a complete protease inhibitor cocktail (EDTA-free; Roche) and phosphatase inhibitor cocktail (Roche). Cells were then sonicated using a Handy Sonic (Tomy Seiko). Protein concentration was determined using the BCA Protein Assay Kit (Thermo Fisher Scientific). Cell lysates were boiled in 1×LDS NuPAGE sample buffer (Thermo Fisher Scientific) for 10 min at 70°C and then electrophoresed on 4–12% NuPAGE Bis-Tris gels (Thermo Fisher Scientific). Proteins were transferred to polyvinylidene difluoride membranes (Millipore). The membranes were blocked in 5% nonfat milk or 3% bovine serum albumin for 1 h at RT and then incubated with primary antibodies for 2 h at RT. The primary antibodies used for immunoblotting were anti-ubiquitin HRP-conjugated mouse monoclonal (sc-8017; Santacruz Biotechnology), anti-K63 ubiquitin rabbit monoclonal (05-1308; clone Apu3; Millipore), anti-K48 ubiquitin rabbit monoclonal (05-1307; clone Apu2; Millipore), anti-USP8 rabbit polyclonal (Kato et al., 2000), anti-TSG101 mouse monoclonal (sc-7964; Santacruz Biotechnology), anti-β-actin HRP-conjugated rabbit polyclonal (PD030; MBL), anti-CCL5 mouse monoclonal (sc-514019; Santacruz Biotechnology), anti-phospho-TAK1 (T184/T187) rabbit polyclonal (4531; Cell Signaling Technology), anti-FLAG HRP-conjugated mouse monoclonal (A8592; Sigma-Aldrich), anti-phospho-p62 (S349) mouse monoclonal (M217-3; clone 5D5; MBL), anti-p62 rabbit polyclonal (PM045; MBL), anti-TAK1 rabbit polyclonal (sc-7162; Santacruz Biotechnology), anti-EEA1 mouse monoclonal (610457; clone 14; BD), anti-GAPDH mouse monoclonal (MAB374; clone 6C5; Millipore), anti-EGFR mouse monoclonal (MI-12-1; clone 6F1; MBL), anti-SP1 rabbit monoclonal (9389; clone D4C3; Cell Signaling Technology), anti-histone H3 rabbit monoclonal (4499; clone D1H2; Cell Signaling Technology), anti-keratin 18 mouse monoclonal (4548; clone DC10; Cell Signaling Technology), anti-multi ubiquitin mouse monoclonal (D058-3; clone FK2; MBL), anti-TAB2 rabbit polyclonal (222214, Abcam), anti-phospho-p38 MAPK (T180/Y182) rabbit polyclonal (9211; Cell Signaling Technology), anti-p38 MAPK (8690; clone D13E1; Cell Signaling Technology), anti-phospho-STAT3 (Y705) rabbit monoclonal (9145; clone D3A7; Cell Signaling Technology), anti-STAT3 rabbit monoclonal (4904; clone 79D7; Cell Signaling Technology), anti-phospho-p44/42 MAPK ERK1/2 (T202/Y204) rabbit monoclonal (4370; clone D13.14.4E; Cell Signaling Technology), anti-p44/42 MAPK ERK1/2 rabbit monoclonal (4695; clone 137F5; Cell Signaling Technology), anti-α-tubulin mouse monoclonal (013-25033; clone 10G10; WAKO), and anti-Myc mouse monoclonal (clone 9E10) antibodies. The membranes were then incubated with secondary antibodies for 50 min at RT. HRP-conjugated goat anti-rabbit Ig and HRP-conjugated goat anti-mouse Ig (Promega) were used as secondary antibodies for immunoblotting. Chemiluminescence images developed in ECL Prime Western Blotting Detection Reagent (GE Healthcare) were acquired using an ImageQuant LAS4000 (GE Healthcare) and a Fusion FX7 (Vilber Bio Imaging). Acquired images were quantified using a FUSION-CAPT (Vilber Bio Imaging).

### TMT 6-plex analysis

Cell lysates were prepared and digested using the EasyPep Mini MS Sample Prep Kit (Thermo Fisher Scientific). 25 µg of peptides from each sample were labeled with 0.2 mg of TMT labeling reagent (Thermo Fisher Scientific) according to the manufacturer’s protocol. After TMT labeling, the sample channels were combined in an equal ratio, dried with a speed-vac, and resuspended in 0.1% TFA. Samples were fractionated into eight fractions using a High pH Reversed-Phase Peptide Fractionation Kit (Thermo Fisher Scientific) according to the manufacturer’s protocol. 1 µg of peptides from each fraction was analyzed by LC-MS/MS on an EASY-nLC 1200-connected Orbitrap Fusion Lumos Tribrid mass spectrometer (Thermo Fisher Scientific) equipped with FAIMS-Pro ion mobility interface (Thermo Fisher Scientific). Peptides were separated on an analytical column (C18, 1.7 µm particle size × 75 µm diameter × 250 mm; IonOpticks) heated at 55°C through a column oven (Sonation) at a constant flow rate of 300 nl/min. Peptides were eluted using a 225-min gradient (3.2–25.6% acetonitrile over 225 min). Peptide ionization was performed using the Nanospray Flex Ion Source (Thermo Fisher Scientific). FAIMS-Pro was set to three phases (−40, −60, and −80 compensation voltage [CV]) with a sec interval, and the Orbitrap Fusion Lumos Tribrid mass spectrometer was operated in the data-dependent acquisition mode using a full scan (m/z range of 375–1,500, nominal resolution of 120,000, target value of 4 × 10^5^ ions) followed by MS/MS scans of the most intense ions in each sec of each CV phase. MS/MS spectra were acquired using a fixed collision energy of 38%, an isolation width of 0.7 m/z, a resolution of 50,000, and a target value of 1 × 10^5^ ions. Precursor ions selected for fragmentation (charge state 2–7) were placed on a dynamic exclusion list for 60 s. MS raw files were analyzed using a Sequest HT search program in Proteome Discoverer 2.4 (Thermo Fisher Scientific). MS/MS spectra were searched against the SwissProt-reviewed human reference proteome (UniProt). TMT-based protein quantification was performed using a Reporter Ions Quantifier node in Proteome Discoverer 2.4.

### qRT-PCR analysis

Total RNA was extracted using Sepasol (Nacalai Tesque) according to the manufacturer’s protocol. cDNA was generated using ReverTra Ace qPCR RT Master Mix with gDNA Remover (TOYOBO) according to the manufacturer’s protocol. Quantification of mRNA was performed with a Light Cycler 480 (Roche) or TP-800 (TAKARA) using THUNDERBIRD SYBR qPCR Mix (TOYOBO). Primer sets used for qRT-PCR analysis were CCL5 forward: 5′-ACC​ACA​CCC​TGC​TGC​TTT​G-3′, CCL5 reverse: 5′-CAC​ACA​CTT​GGC​GGT​TCT​TTC-3′, SOD2 forward: 5′-TGG​AAG​CCA​TCA​AAC​GTG​AC-3′, SOD2 reverse: 5′-AAA​CCA​AGC​CAA​CCC​CAA​C-3′, HO1 forward: 5′-CTT​TCA​GAA​GGG​CCA​GGT​G-3′, HO1 reverse: 5′-GGA​AGT​AGA​CAG​GGG​CGA​AG-3′, and actin forward: 5′-TCC​CTG​GAG​AAG​AGC​TAC​GAG-3′, actin reverse: 5′-GGA​AGG​AAG​GCT​GGA​AGA​GTG-3′.

### Luciferase assay

The human *CCL5* promoter was amplified from genomic DNA of HEK 293 cells and inserted into pNL1.2 (Promega). Mutant *CCL5* promoters lacking NF-κB binding sites were generated by overlap PCR with nucleotide substitution by adenines. Plasmids were transfected using polyethylenimine. Cells transfected with pNL1.2 together with the control pGL4.53 or pGL4.54 vector (Promega) were assayed using a Nano-Glo Dual-Luciferase Reporter Assay System (Promega) according to the manufacturer’s protocol.

### Statistical analysis

Statistical analysis was performed with GraphPad Prism 8. All statistical information is described in the figure legends. First, the sample distribution was assessed using D’Agostino–Pearson, Shapiro–Wilk, or Kolmogorov–Smirnov normality tests depending on the sample size. Data with small sample sizes were assumed to have a normal distribution but these were not formally tested. The unpaired two-tailed Student’s *t* test was used to determine statistical significance when comparing unpaired two independent groups with normal distribution and no significant difference in their standard deviation (SD). Ordinary one-way ANOVA with Dunnett’s multiple comparison test was used for multiple comparisons involving more than two unpaired groups with normal distribution. Kruskal–Wallis and Dunn’s post-hoc tests were used for multiple comparisons of more than two unpaired groups where at least one group was not normally distributed. In all cases, statistical significance was assessed with a 95% confidence interval, and a P value <0.05 was considered significant.

### Online supplemental material

Fig. S1 shows the endosomal ubiquitin accumulation caused by the reduction of USP8 deubiquitinating activity. Fig. S2 shows proteomic analysis of USP8- and TSG101-depleted cells. Fig. S3 shows TAB2/3-mediated signal transduction in USP8-depleted cells. Fig. S4 shows the accumulation of phosphorylated p62 on endosomes in USP8-depleted cells. Fig. S5 shows the colocalization of p62 and Keap1 on endosomes in USP8-depleted cells. Table S1 lists the ubiquitinated proteins identified by MS analysis. Table S2 lists the ubiquitinated proteins significantly increased in USP8-depleted cells. Table S3 contains proteome analysis of USP8- and TSG101-depleted cells.

## Supplementary Material

Table S1 shows a list of ubiquitinated proteins identified by MS analysis.

Table S2 shows a list of ubiquitinated proteins significantly increased in USP8-depleted cells.

Table S3 shows proteome analysis of USP8- and TSG101-depleted cells.

SourceData F2 is the source file for Fig. 2.

SourceData F3 is the source file for Fig. 3.

SourceData F4 is the source file for Fig. 4.

SourceData FS1 is the source file for Fig. S1.

SourceData FS2 is the source file for Fig. S2.

SourceData FS3 is the source file for Fig. S3.

SourceData FS4 is the source file for Fig. S4.

SourceData FS5 is the source file for Fig. S5.

## Data Availability

The mass spectrometry proteomics data have been deposited to the ProteomeXchange Consortium via the PRIDE partner repository with dataset identifiers PXD042488 (related to Fig. 1, D–G) and PXD042490 (related to Fig. 2, B–E).
