# Supplementary figures and images for: USP8 prevents aberrant NF-κB and Nrf2 activation by counteracting ubiquitin signals from endosomes
Source: J Cell Biol. 2024 Jan 5;223(3):e202306013. doi: 10.1083/jcb.202306013 (PMC10783432; doi:10.1083/jcb.202306013)

Figure 2A

Ubiquitin (pan)

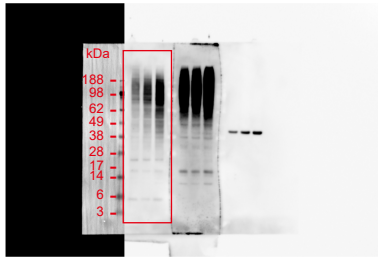

Ubiquitin (K63)

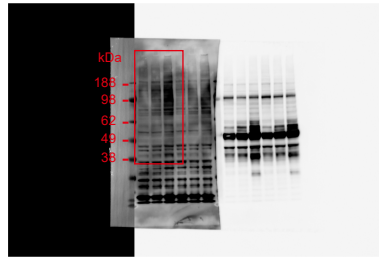

Ubiquitin (K48)

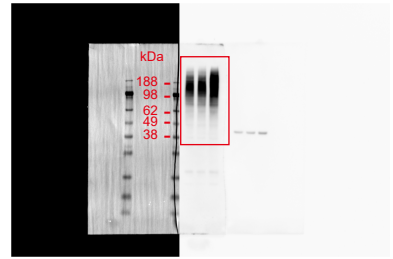

USP8

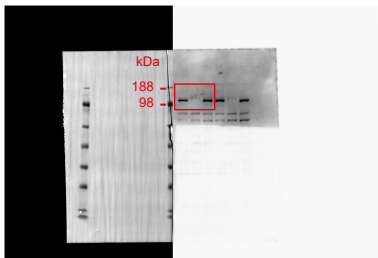

TSG101

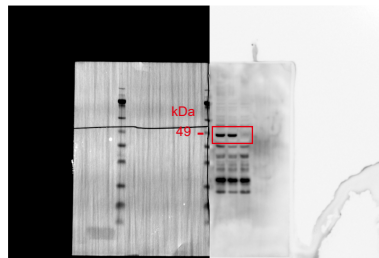

Actin

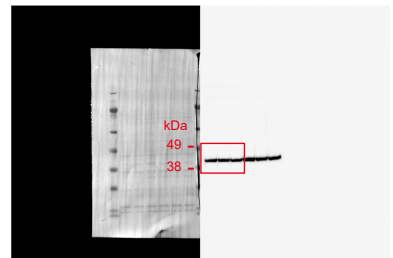

Figure 2F

CCL5

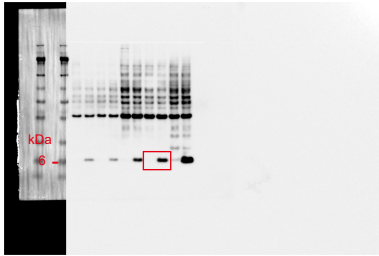

USP8

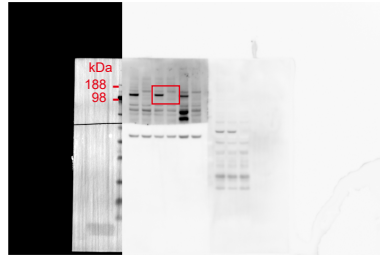

Actin

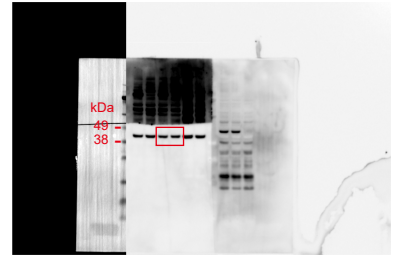

Supplement: SourceData F2 — is the source file for Fig. 2. [file JCB_202306013_SourceDataF2.pdf]

Figure 3D

phospho-TAK1(T184/187)

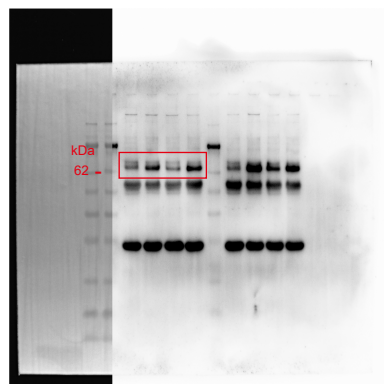

FLAG

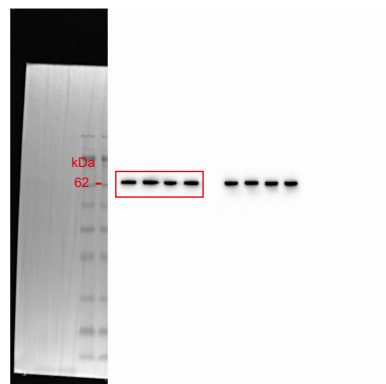

FLAG

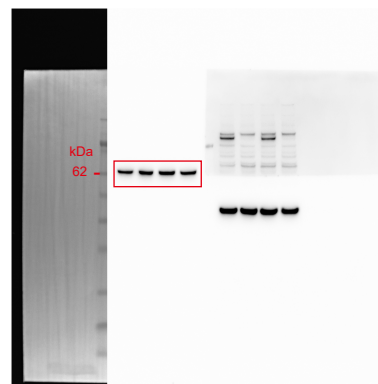

USP8

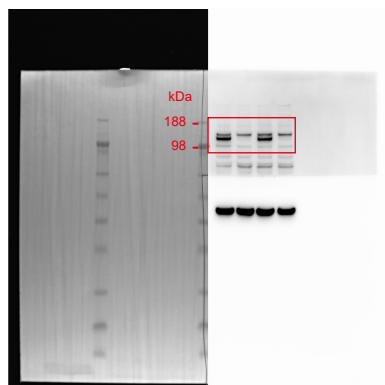

Actin

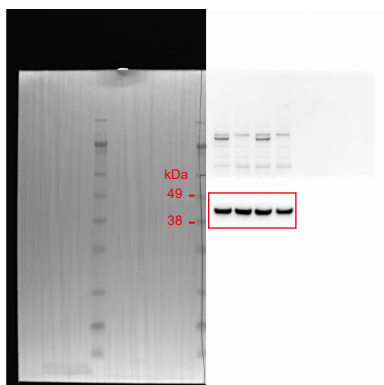

Supplement: SourceData F3 — is the source file for Fig. 3. [file JCB_202306013_SourceDataF3.pdf]

Figure 4A

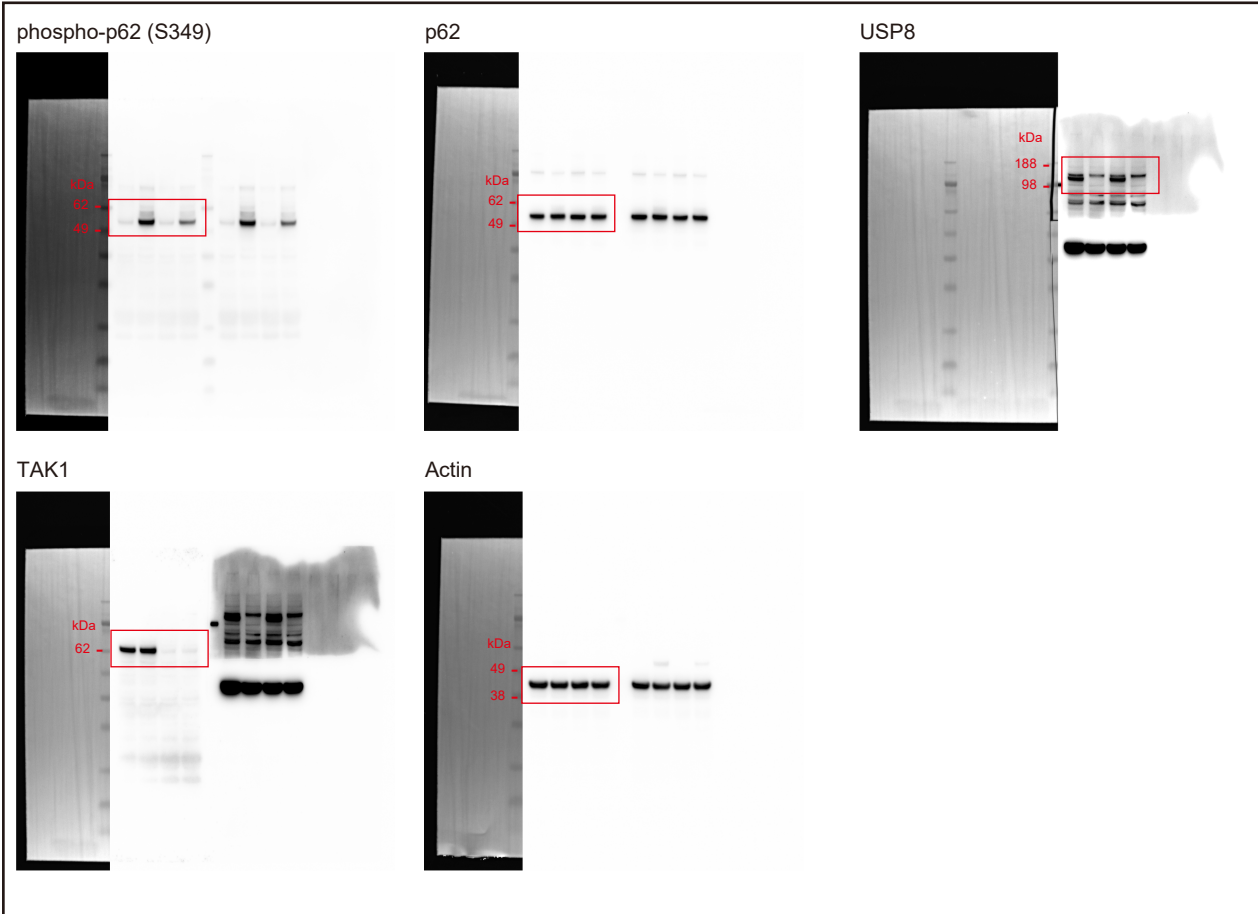

Supplement: SourceData F4 — is the source file for Fig. 4. [file JCB_202306013_SourceDataF4.pdf]

Figure S1A

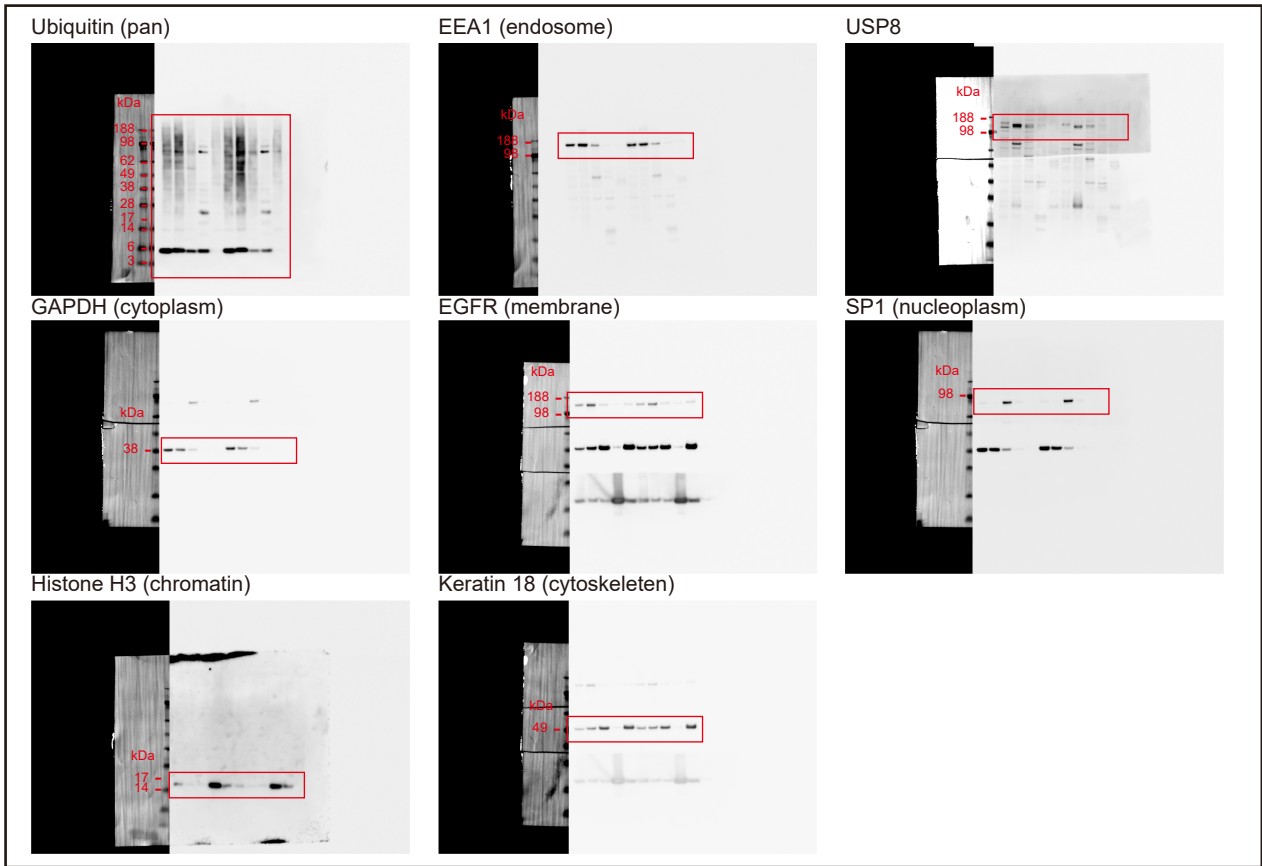

Figure S1E

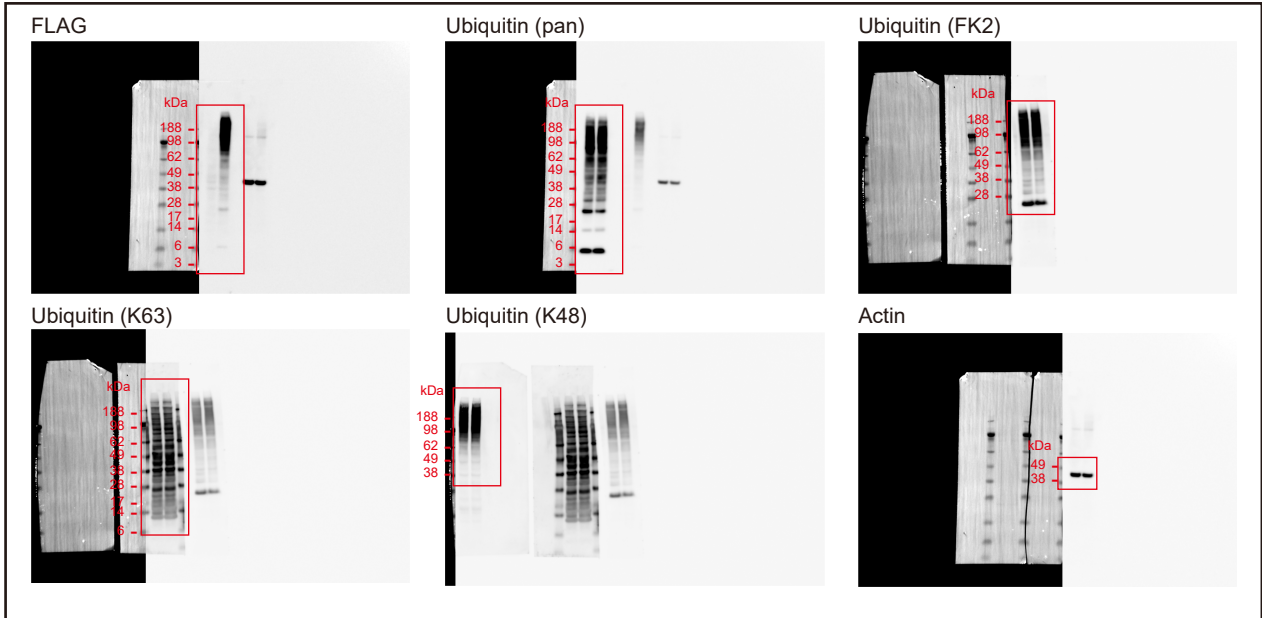

Supplement: SourceData FS1 — is the source file for Fig. S1. [file JCB_202306013_SourceDataFS1.pdf]

Figure S2C

CCL5

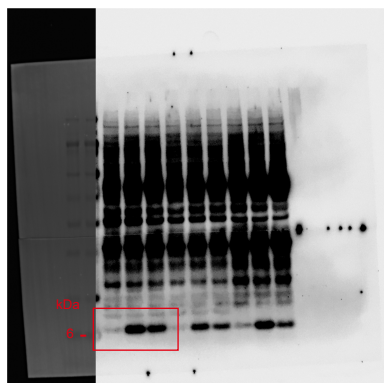

USP8

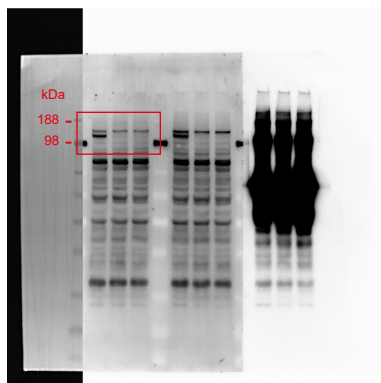

Actin

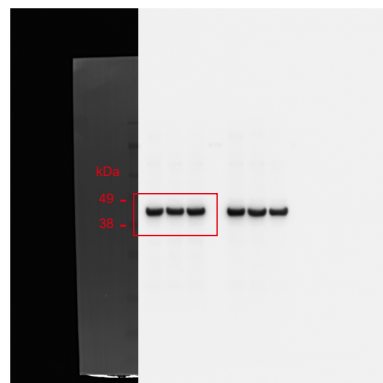

Supplement: SourceData FS2 — is the source file for Fig. S2. [file JCB_202306013_SourceDataFS2.pdf]

Figure S3C

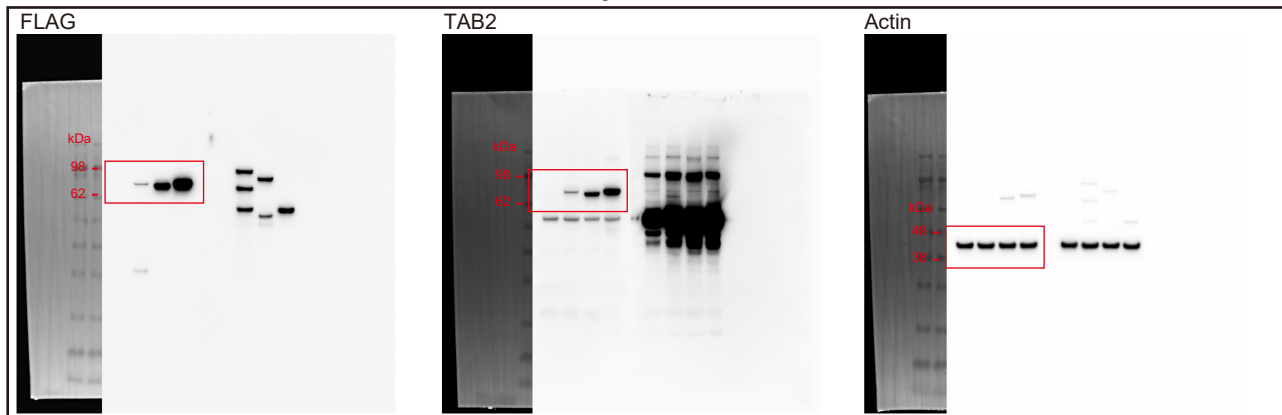

Figure S3G

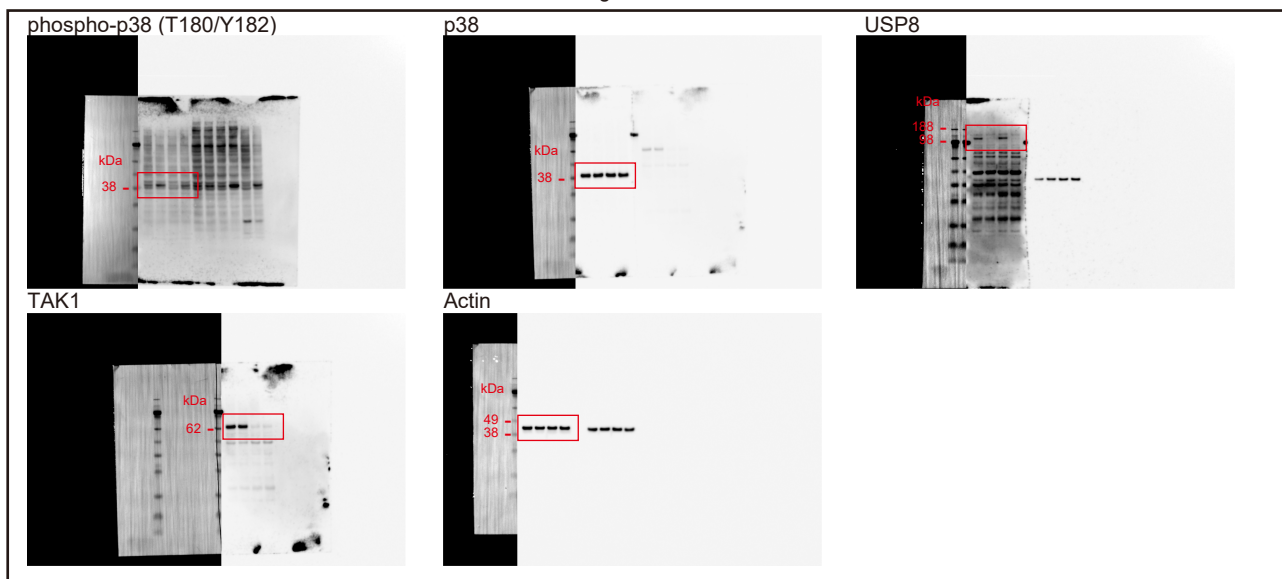

Figure S3H

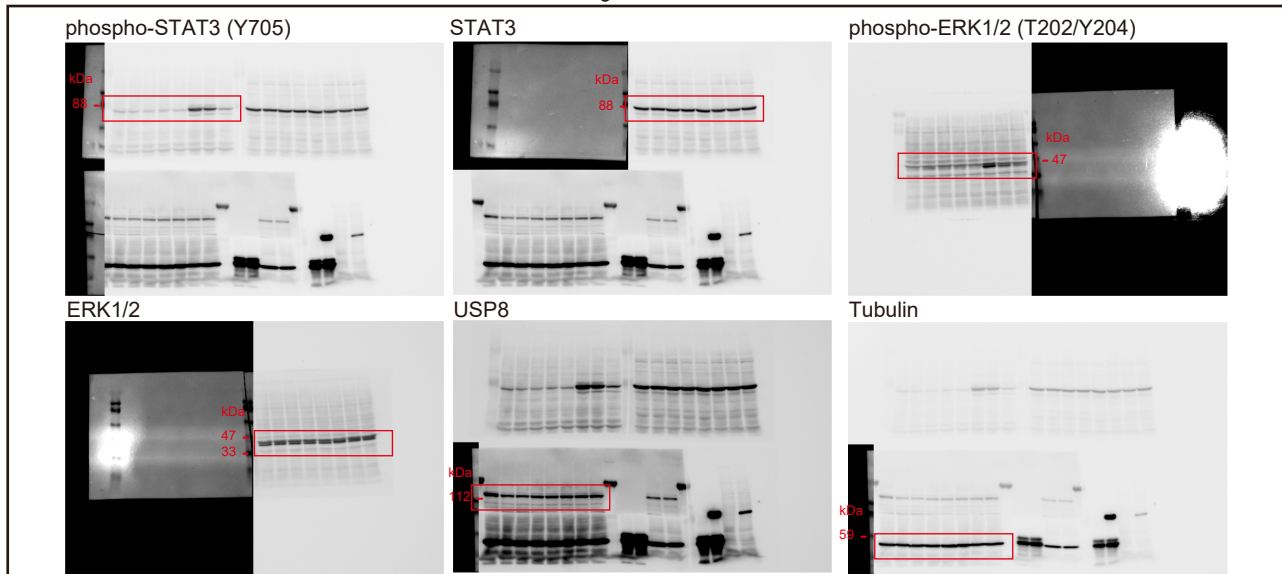

Supplement: SourceData FS3 — is the source file for Fig. S3. [file JCB_202306013_SourceDataFS3.pdf]

Figure S4A

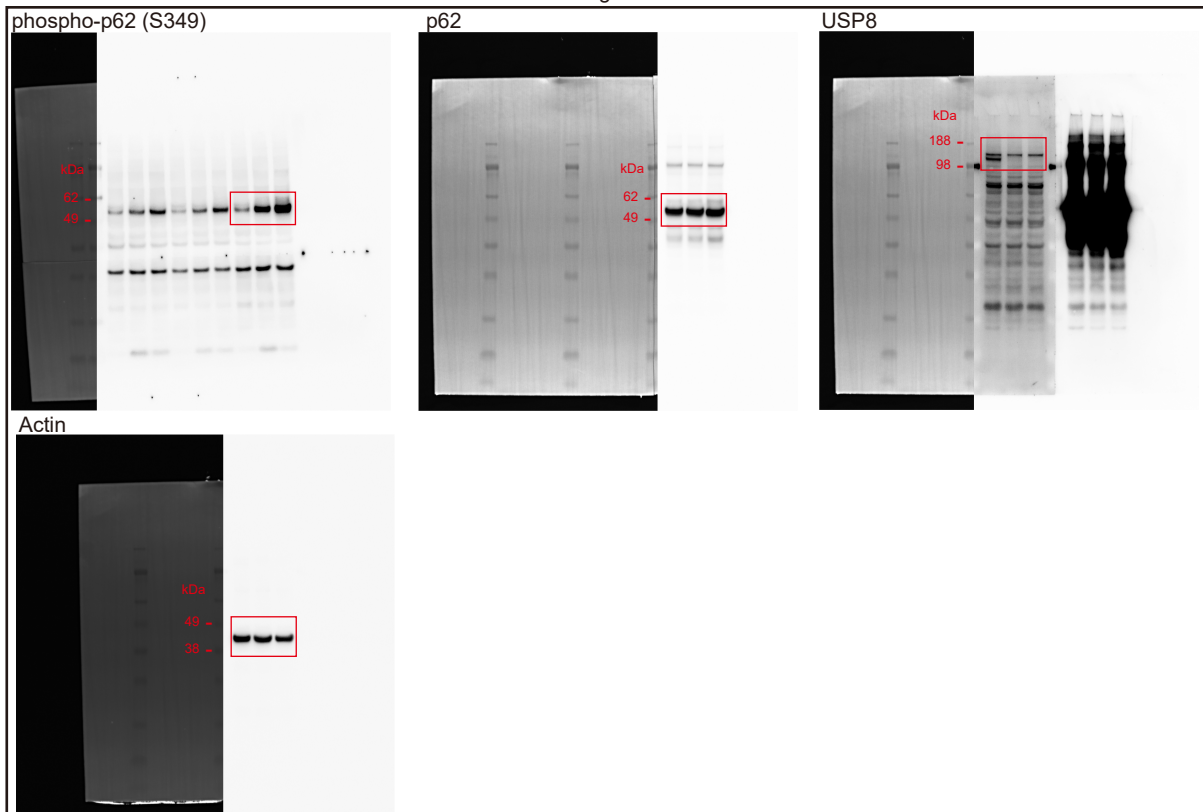

Figure S4D

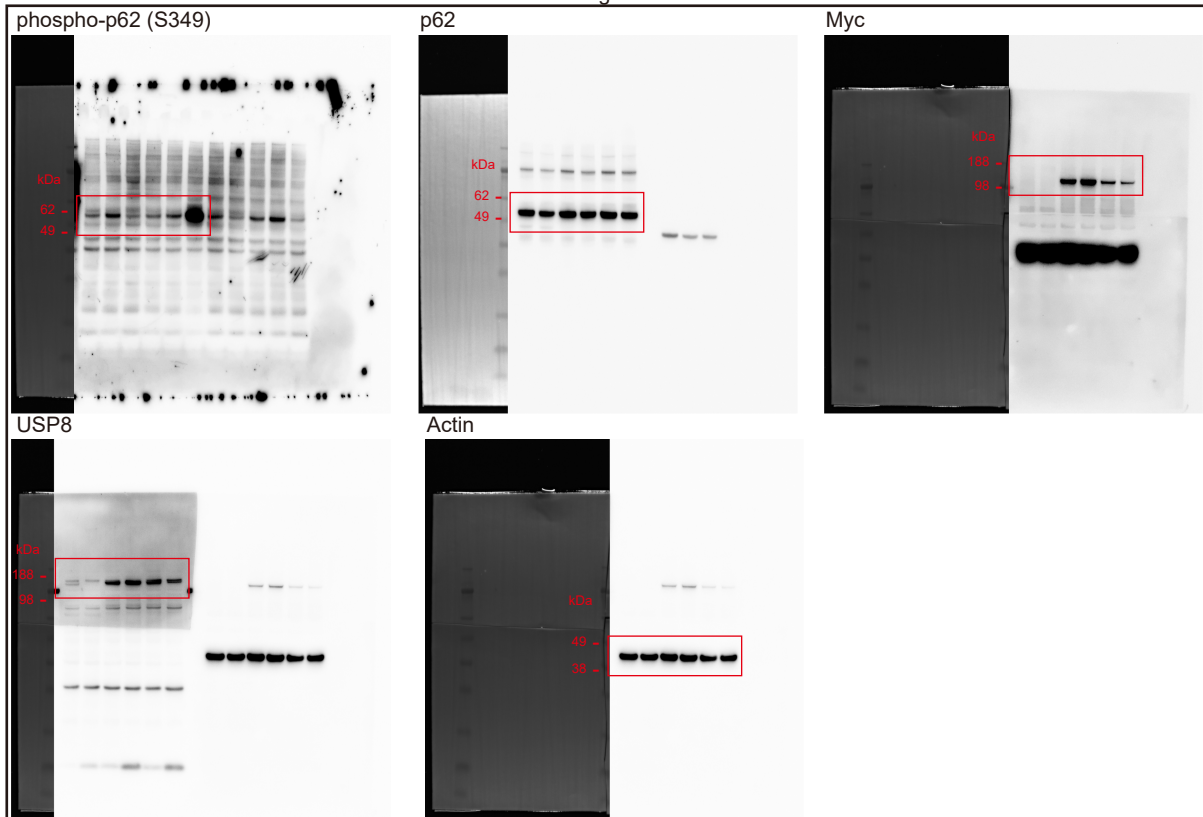

Supplement: SourceData FS4 — is the source file for Fig. S4. [file JCB_202306013_SourceDataFS4.pdf]

Figure S5A

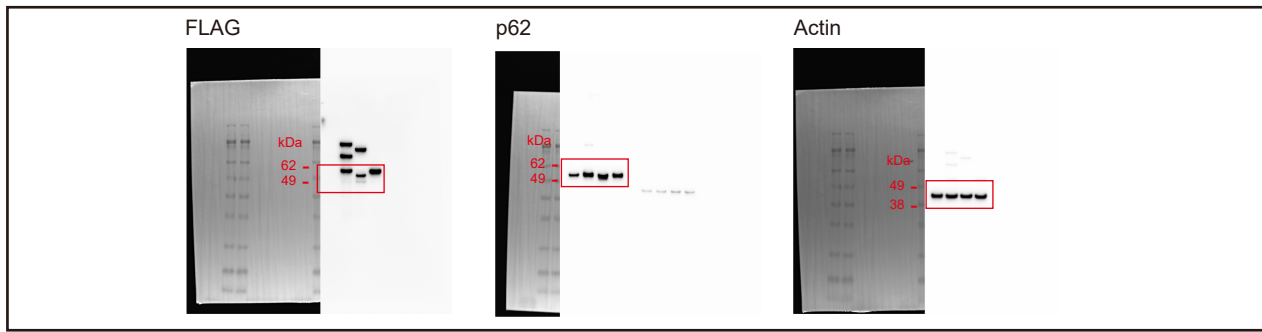

Figure S5C

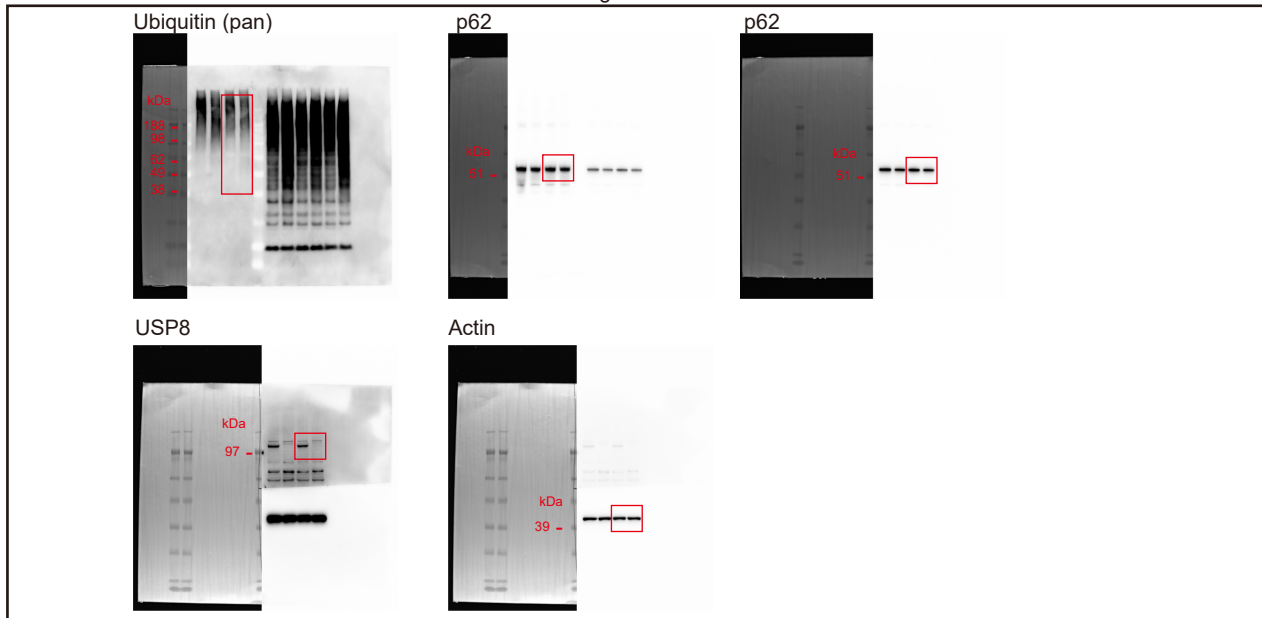

Figure S5D

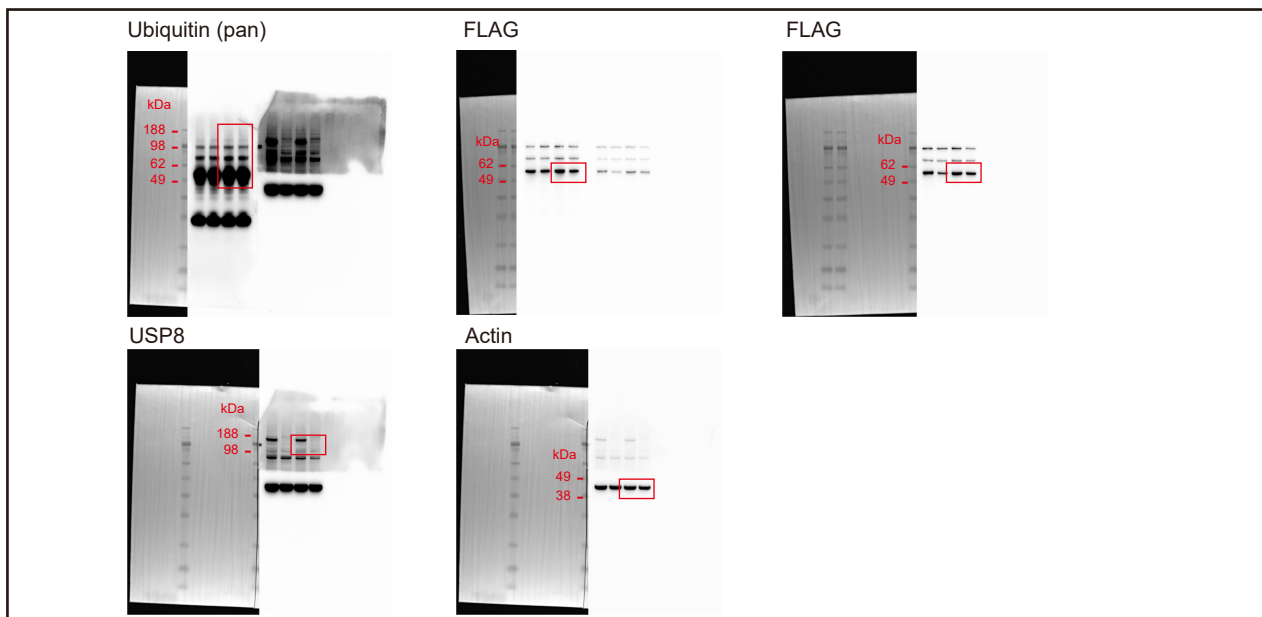

Supplement: SourceData FS5 — is the source file for Fig. S5. [file JCB_202306013_SourceDataFS5.pdf]
